# Supplementary material for: Chemical modification of proteins by insertion of synthetic peptides using tandem protein trans-splicing
Source: Nat Commun. 2020 May 8;11:2284. doi: 10.1038/s41467-020-16208-6 (PMC7210297; doi:10.1038/s41467-020-16208-6)
Supplement: Supplementary file 1 — Supplementary Information [file 41467_2020_16208_MOESM1_ESM.pdf]

Supplementary Information

Chemical modification of proteins by insertion of synthetic peptides using tandem protein trans-splicing

Khoo, K.K. and Galleano, I. et al.

Supplementary Figures 1-13
Supplementary Tables 1-2
Supplementary Methods
Supplementary References

Supplementary Figures

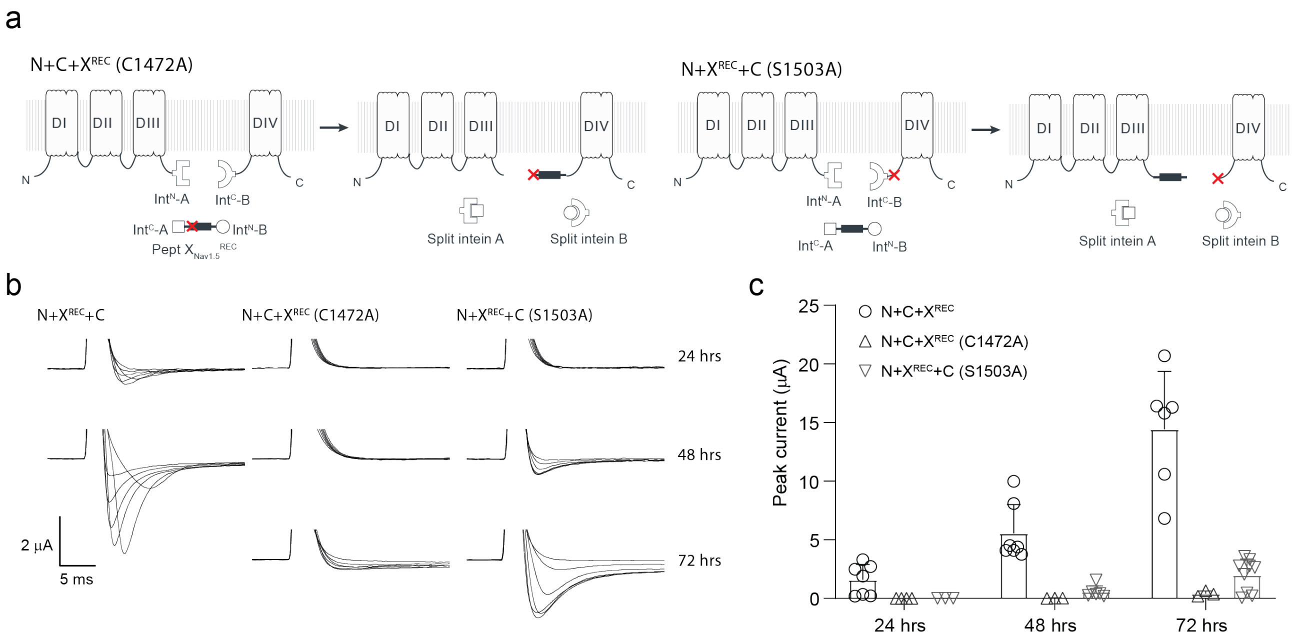

Supplementary figure 1: Control experiments for tPTS in Na$_V$1.5 DIII-DIV linker (recombinant expression). (a) Schematic presentation of non-spliced Na$_V$1.5 fragments expected for the Na$_V$1.5 DIII-DIV linker splice sites tested when +1 extein residues of each split intein is mutated to alanine (indicated by red cross): C1472A in intein A and S1503A in intein B. This prevents splicing and favors side reactions, resulting in accumulation of cleavage products (note that this will likely overrepresent the occurrence of the side reactions compared to when splicing-competent split inteins are used, i.e. Fig 1). (b) Representative families of sodium current traces in response to voltage steps from -50 mV to +10 mV in 10 mV steps, recorded 24, 48, or 72 hrs after mRNA injection of N+X^{REC}+C. Note that oocytes with peak currents exceeding 2 µA are typically not ideally voltage-clamped, which decreases the accuracy of the values obtained. As expected, functional non-spliced constructs (e.g. N+X^{REC}+C [S1503A]) display currents with impaired (slower) inactivation. (c) Average peak currents recorded for each combination depicted as a bar plot (mean +/- SD; n = 3-9, as indicated by data dot plots) for the respective time points (note that experiments shown in Fig 1 were conducted 24 hours after N+C mRNA injection or 12-20 hrs after injection of peptide X^{SYN}). Source data are provided as a Source Data file.

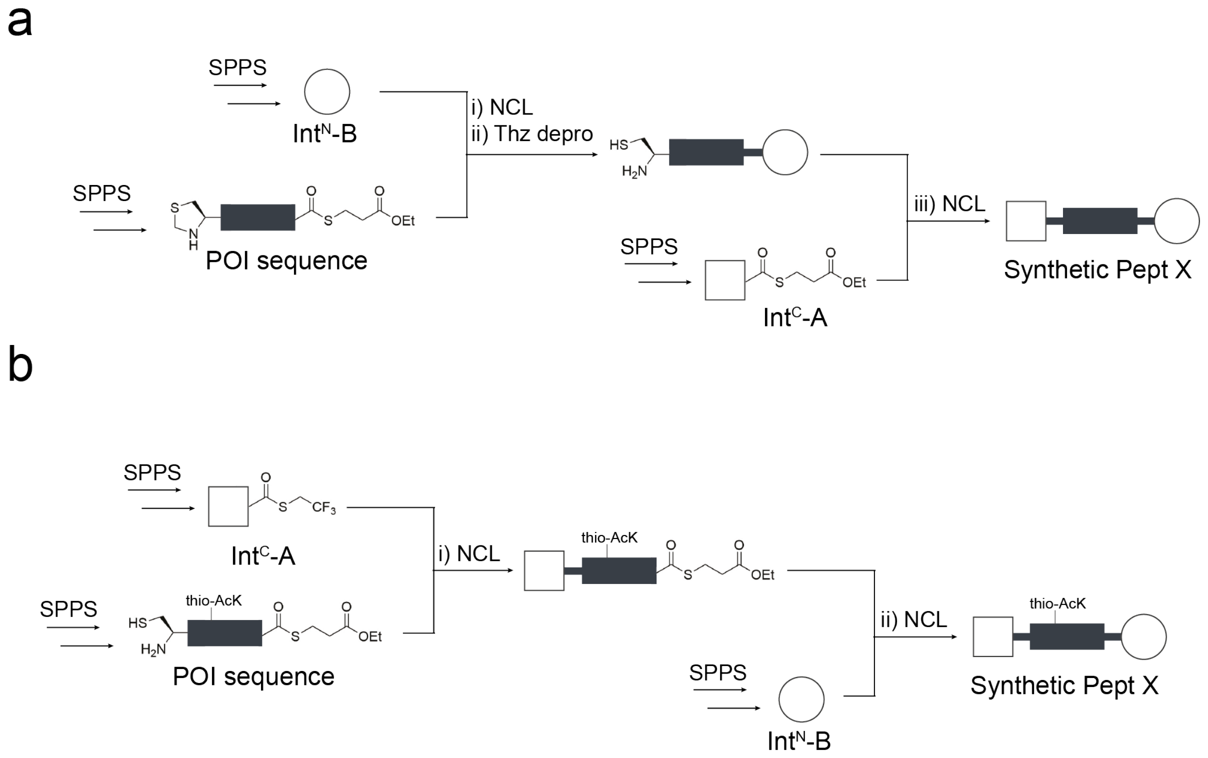

Supplementary figure 2: Peptide X synthesis strategies. (a) C-to-N 'one-pot' ligation of three parts, which were synthesized by standard SPPS. The two ligation steps were performed by exploiting the standard cysteine Thz-masking approach. (b) For peptides X containing a thio-acetylated lysine residue, an alternative N-to-C 'one-pot' ligation was adopted. This strategy exploited the faster kinetics of the TFET thioester of Int^C-A compared to the alkyl thioester of the POI peptide, allowing us to avoid orthogonal protecting groups, e.g. Thz.

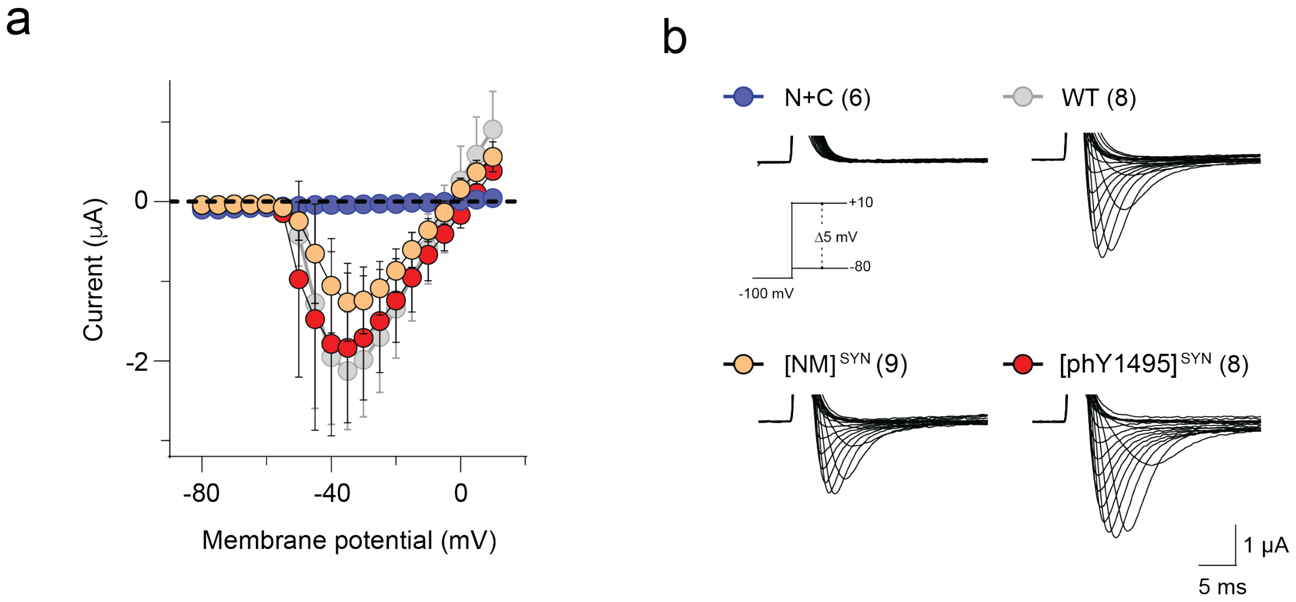

Supplementary figure 3: Spliced Na$_V$1.5 channels containing synthetic peptide X showed depolarization-evoked currents of amplitudes comparable to WT Na$_V$1.5. (a) Current-voltage relationships and (b) representative sodium currents of *Xenopus laevis* oocytes expressing N and C constructs only (N+C), Na$_V$1.5 (WT), N+C+X^{NM} ([WT^{NM}]^{SYN}) and N+C+X^{phY1495} ([phY1495]^{SYN}) in response to depolarizing voltage steps (-80 to +10 mV in 5 mV steps with a holding potential of -100 mV). *Xenopus laevis* oocytes were injected with RNAs of N+C or WT Na$_V$1.5 48 hours before recording, whereas synthetic peptides X (X^{NM} or X^{phY1495}) were injected into N+C RNA-injected oocytes 24 hours before recording. To ensure adequate control of voltage clamp, [Na$^+$] in the extracellular recording solution was reduced, (in mM): 24 NaCl, 72 NMDG, 2 KCl, 1.8 $CaCl_2$, 1 $MgCl_2$ and 5 HEPES, pH 7.4 with HCl. Data in (a) are shown as mean ± SD; n=6-9. Numbers in parentheses in (b) indicate number of individual cells used for recordings. Source data are provided as a Source Data file.

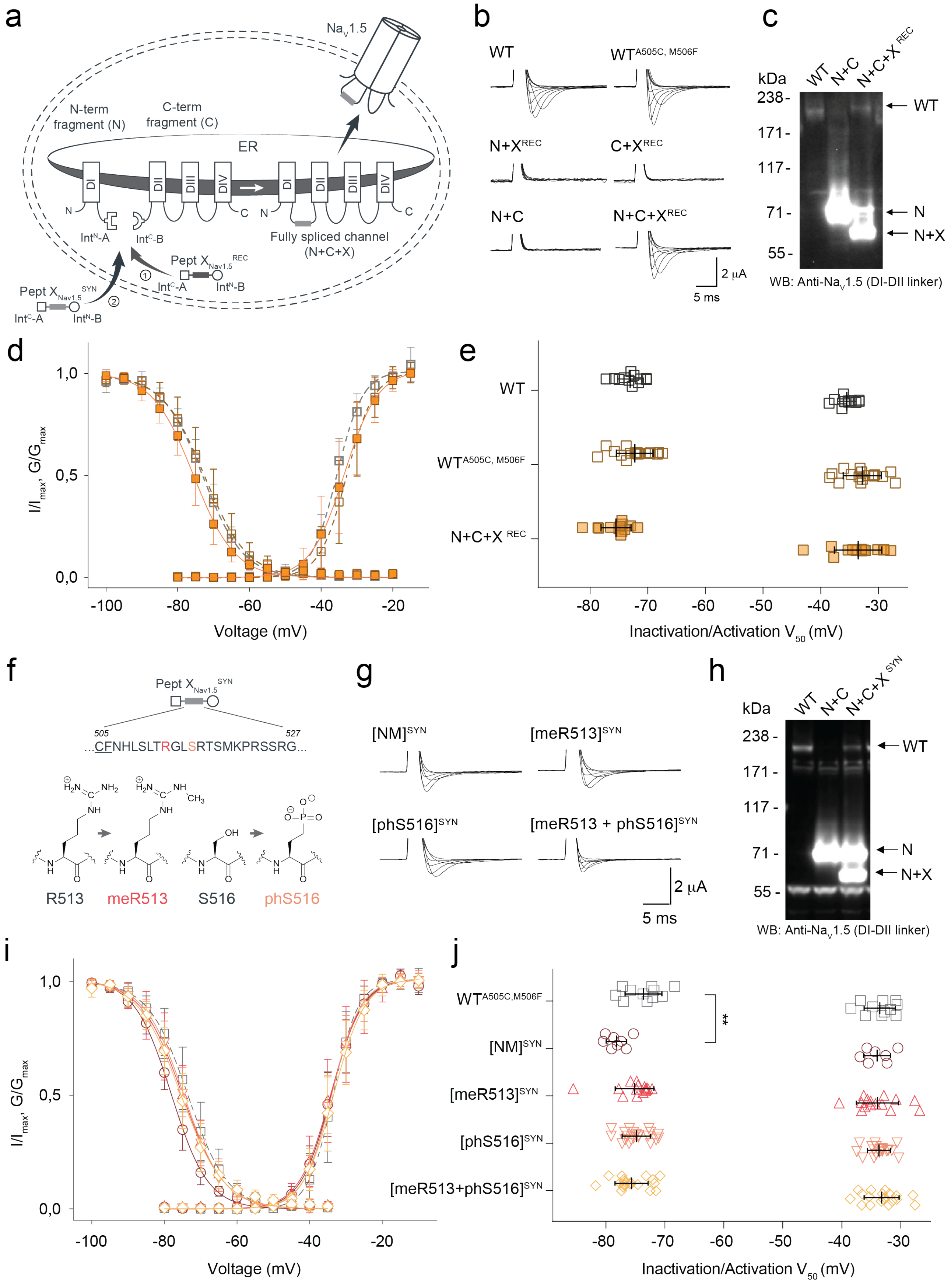
a
NaV1.5
N-term fragment (N)
C-term fragment (C)
ER
DI
DII
DIII
DIV
Fully spliced channel (N+C+X)
Pept X Nav1.5 REC
Pept X Nav1.5 SYN
IntC-A
IntN-B
IntN-A
IntC-B
b
WT
WTA505C, M506F
N+XREC
C+XREC
N+C
N+C+XREC
2 μA
5 ms
c
kDa
WT
N+C
N+C+X REC
238
171
117
71
55
WT
N
N+X
WB: Anti-NaV1.5 (DI-DII linker)
d
I/Imax, G/Gmax
1,0
0,5
0,0
-100
-80
-60
-40
-20
Voltage (mV)
e
WT
WTA505C, M506F
N+C+X REC
-80
-70
-60
-50
-40
-30
Inactivation/Activation V50 (mV)
f
Pept X Nav1.5 SYN
505
527
...CFNHLSLTRGLSRTSMKPRSSRG...
R513
meR513
S516
phS516
g
[NM]SYN
[meR513]SYN
[phS516]SYN
[meR513 + phS516]SYN
2 μA
5 ms
h
kDa
WT
N+C
N+C+X SYN
238
171
117
71
55
WT
N
N+X
WB: Anti-NaV1.5 (DI-DII linker)
i
I/Imax, G/Gmax
1,0
0,5
0,0
-100
-80
-60
-40
-20
Voltage (mV)
j
WTA505C,M506F
[NM]SYN
[meR513]SYN
[phS516]SYN
[meR513+phS516]SYN
**
-80
-70
-60
-50
-40
-30
Inactivation/Activation V50 (mV)

Supplementary figure 4: tPTS to insert recombinant or synthetic peptides into the Na_V1.5.DI-DII linker. (a) Schematic overview of the applied approach using either recombinant (strategy 1) or synthetic peptide $X_{Nav1.5}^{SYN}$ (corresponding to amino acids 505 to 527 of the Na_V1.5 DI-DII linker; strategy 2) to reconstruct full-length Na_V1.5 from recombinantly expressed N-/C-terminal fragments (N and C) in *Xenopus laevis* oocytes. Inteins A (*Cfa*DnaE) and B (*Ssp*DnaB^{M86}) are indicated by square and round symbols, respectively. (b) Representative sodium currents in response to sodium channel activation protocol (see methods; only voltage steps from -50 mv to +10 mV in 10 mV steps are displayed), demonstrating expression of functional Na_V1.5 only in the presence of all three recombinantly expressed components (N+X^{REC}+C), along with WT and A505C, M506F double mutant channels (introduced to create a functional splice site for intein A). (c) Western blot analysis verifying presence of fully spliced Na_V1.5 only when all three components (N+X^{REC}+C) were co-expressed (using antibody against Na_V1.5 DI-DII linker residues 493-511). (d) Steady-state inactivation and conductance-voltage (G-V) relationships for respective full-length and spliced constructs. (e) Comparison of values for half-maximal (in)activation (V_{50}) (values are displayed as mean +/- SD; WT, n = 12; $WT^{A505C,M506F}$, n = 17; N+C+X^{REC}, n = 14). (f) Sequence of $X_{Nav1.5}$ corresponding to the amino acids replaced in the Na_V1.5 DI-DII linker and chemical structures of native amino acids and PTMs incorporated into the respective positions of the DI-DII intracellular linker via chemical synthesis of peptide $X_{Nav1.5}^{SYN}$. Note that a non-hydrolysable phosphonylated serine was used (phS). Underlined residues indicate A505C, M506F mutations that were introduced to optimize the splicing reaction. (g) Representative sodium currents in response to voltage steps from -50 mV to +10 mV in 10 mV steps, demonstrating expression of functional Na_V1.5 when *Xenopus laevis* oocytes expressing N and C constructs were injected with synthetic peptides containing non-modifiable side chains in positions 513 and 516 (R513K and S516V, NM), meR513 or phS516 or both PTMs together. (h) Immunoblot verifying presence of fully spliced Na_V1.5 only when synthetic peptide $X_{Nav1.5}^{SYN}$ was injected into cells expressing N and C-terminal fragments (using antibody against Na_V1.5 DI-DII linker residues 493-511). (i) Steady-state inactivation and conductance-voltage (G-V) relationships for PTM- modified/non-modified constructs. (j) Comparison of values for half-maximal (in)activation (V_{50}) (values are displayed as mean +/- SD; $WT^{A505C,M506F}$, n = 11; NM, n = 8; meR513, n = 15; phS516, n = 21; meR513+phS516, n = 21). Significant differences were determined by one-way ANOVA with a Tukey post-hoc test. **, p<0.003 ($WT^{A505C,M506F}$ vs NM, p=0.0056). Source data are provided as a Source Data file.

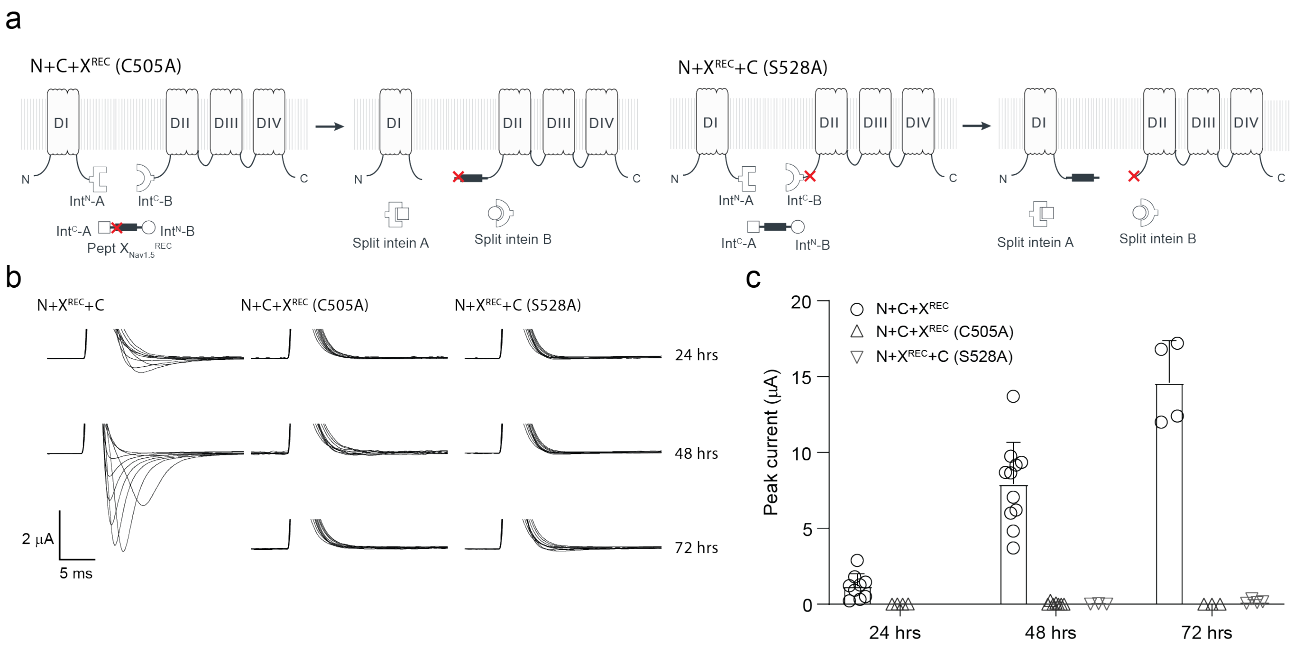

Supplementary figure 5: Control experiments for tPTS in $Na_V1.5$ DI-DII linker (recombinant expression). (a) Schematic presentation of non-spliced $Na_V1.5$ fragments expected for the $Na_V1.5$ DI-DII linker splice sites tested when +1 extein residues of each split intein is mutated to alanine (indicated by red cross): C505A in intein A and S528A in intein B. This prevents splicing and favors side reactions, resulting in cleavage products to accumulate (note that this will likely overrepresent the occurrence of the side reactions compared to when splicing-competent split inteins are used, i.e. Supplementary Fig 4). (b) Representative sodium current traces in response to voltage steps from -50 mV to +10 mV in 10 mV steps, recorded 24, 48 or 72 hours after mRNA injection of N+X^{REC}+C. Note that oocytes with peak currents exceeding 2 μA are typically not ideally voltage-clamped, which decreases the accuracy of the values obtained. (c) Average peak currents recorded for each combination depicted as a bar plot (mean +/- SD; n= 3-11, as indicated by data dot plots) for the respective time points (note that experiments shown in Supplementary Fig 4 were conducted 24 hrs after mRNA injection or 12-20 hrs after injection of peptide $X_{Nav1.5}^{SYN}$). Source data are provided as a Source Data file.

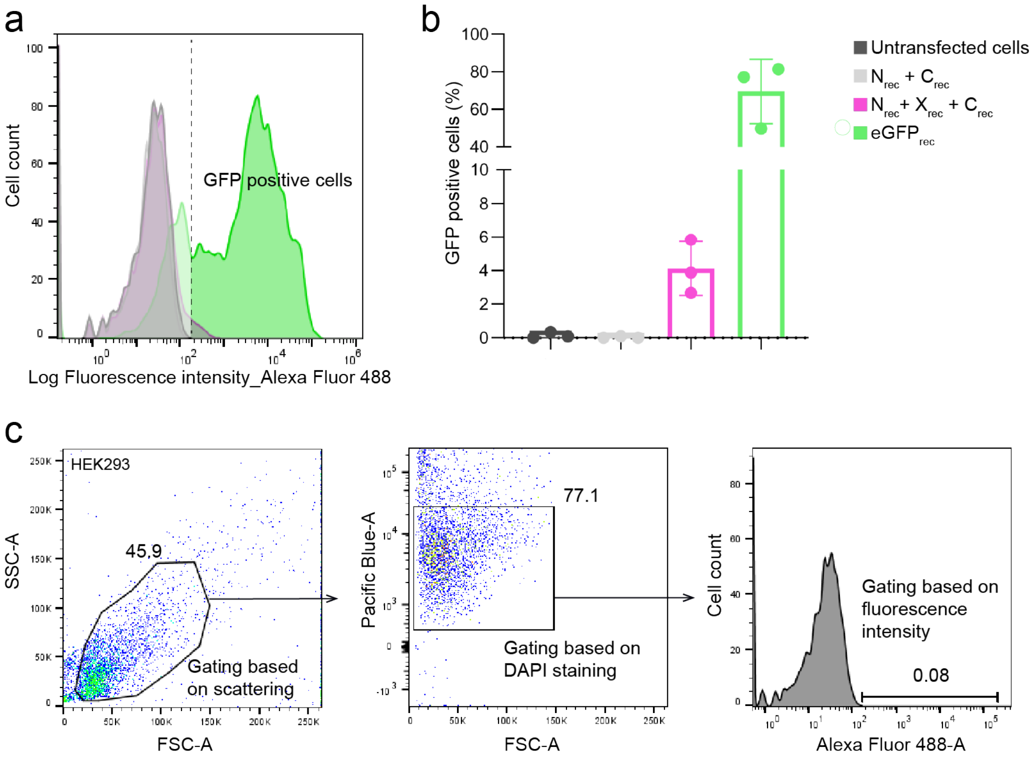

Supplementary figure 6: FACS analysis of reconstituted eGFP using cytosolic tPTS in mammalian cells. HEK293 cells were transfected with DNA coding for N- or C-terminal GFP fragments only (N_{rec} + C_{rec}; grey) or together with peptide X_{GFP}^{REC} (N_{rec} + X_{rec} + C_{rec}; pink). Cellular fluorescence was analyzed and compared to that of untransfected cells (black) and eGFP transfected cells (green) using a BD™ LSR II flow cytometer. (a) Representative histogram plot of the fluorescence distribution from the analyzed cells. The fluorescence intensity cut off determining GFP positive cells (indicated by the dashed line) was set at the upper limit of fluorescence distribution from the untransfected cell population (<0.1% of untransfected cells fall in this area). (b) Bar graph showing % of GFP positive cells for each combination represented (mean +/- SD; n= 3). (c) Illustration of gating strategy applied to determine the 'GFP positive' cell population. The gating strategy was first created based on the untransfected sample and then applied to the other samples from the same batch.

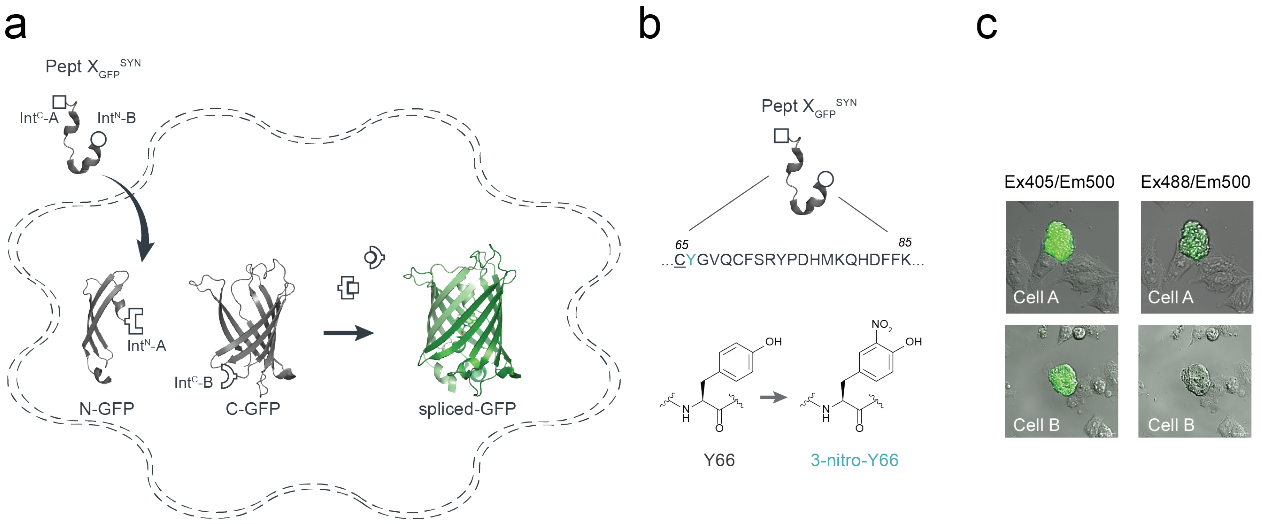

Supplementary figure 7: Insertion of synthetic peptide X_{GFP}^{SYN} into GFP using cytosolic tPTS in mammalian cells. (a) Schematic presentation of the strategy applied to reconstitute GFP from recombinantly expressed N-/C-terminal fragments and synthetic peptide X_{GFP}^{SYN} corresponding to amino acids 65-85 of GFP in HEK293 cells. Inteins A (*Cfa*DnaE) and B (*Ssp*DnaB^{M86}) are indicated by square and round symbols, respectively. (b) Sequence of peptide X_{GFP}^{SYN} corresponding to the amino acids replaced in GFP and chemical structures of tyrosine and its ncAA derivative (3-nitro-tyrosine) incorporated into position66 within the GFP chromophore via chemical synthesis of peptide X_{GFP}^{SYN}. (c) Two examples (cell A and B, respectively) of overlaid brightfield and fluorescence images of HEK293 cells transfected with N and C fragments and squeezed in the presence of peptide 20 µM X containing 3-nitro-Tyr in position 66. Note the brighter fluorescence emission when cells were excited with a 405-nm laser compared to excitation with a 488-nm laser, indicating a blue-shift in the spectral properties of the 3-nitro-tyrosine-containing GFP variant. Scale bars: 20 µm.

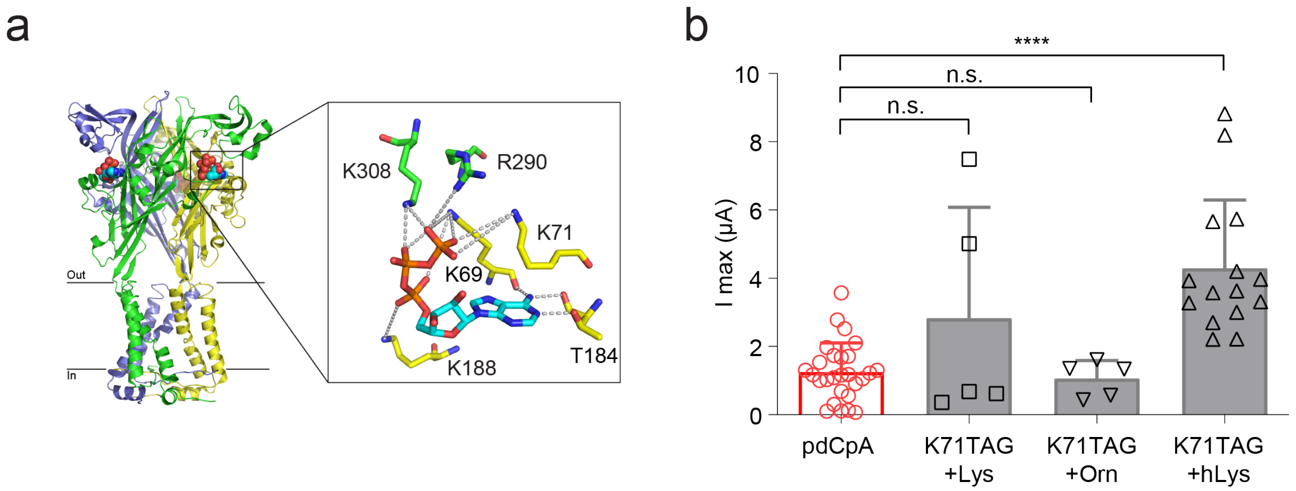

Supplementary figure 8: Incorporation of lysine derivatives in K71 position of the P2X2R using ribosome based non-sense suppression method in *Xenopus laevis* oocytes. (a) Crystal structure of ATP-bound hP2X3R (PDB: 5svk). Inset highlights interaction of K71 (P2X2 residue numbering) with the phosphate tail of ATP. (b) Peak currents elicited by 50 mM ATP at oocytes injected with uncharged tRNA (pdCpA, red bar) or mRNA coding for K71TAG with lysine, ornithine (Orn) or homolysine (hLys) charged tRNAs. Non-significant differences in peak current size recorded from oocytes injected with uncharged and charged tRNAs (with the exception of hLys) indicate possible non-specific incorporation of endogenous amino acids[1] at the K71TAG site. Values are depicted as a bar plot (mean +/- SD; pdCpA, n =27; K71TAG+Lys, n = 5; K71TAG+Orn, n = 5; K71TAG+hLys, n = 17). Significant differences were determined by one-way ANOVA with a Tukey post-hoc test. n.s., $p>0.03$ (pdCpA vs K71TAG+Lys, $p=0.4688$; pdCpA vs K71TAG+Orn, $p=0.9997$); ****, $p<0.0001$. Source data are provided as a Source Data file.

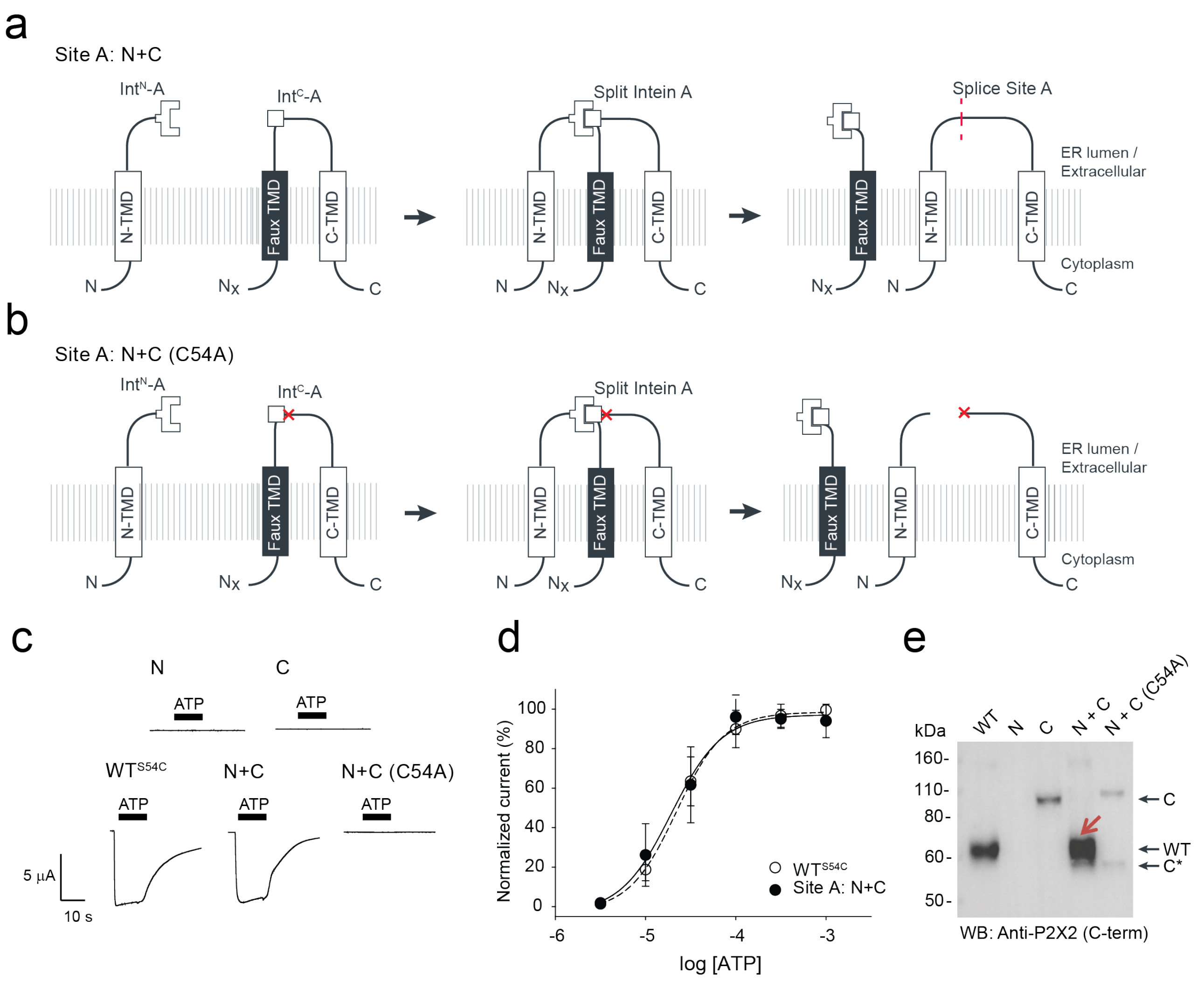

Supplementary figure 9: Single-intein PTS of the P2X2R extracellular domain at splice site A. (a) Schematic presentation of single-intein PTS at position 54 (site A) in the extracellular domain of P2X2Rs in *Xenopus laevis* oocytes. Note that a *faux* transmembrane helix was engineered into the C-terminal fragment to maintain its native membrane topology during protein expression. S54C mutation (at splice site A in P2X2) was introduced in the C-terminal fragment to create an optimized splice site for intein A. Intein A (*Cfa*DnaE) is indicated by square symbols. (b) Schematic presentation of non-spliced P2X2R fragments expected when +1 extein residue of split intein A (position 54 of P2X2) is mutated to alanine (indicated by red cross). This prevents splicing and favors side reactions, resulting in cleavage products to accumulate (note that this will likely overrepresent the occurrence of the side reactions compared to when splicing-competent split inteins are used). (c) Representative current traces during application of ATP (300 μM, indicated by black bars) to oocytes expressing respective constructs recorded one day after injection of mRNA. ATP-induced currents were absent under control conditions even after multiple days after injection. (d) Concentration-response curve (CRC) of reconstituted receptor indicates wild-type like functionality. Values are displayed as mean +/- SD; WT^{S54C}, n = 5; N+C, n = 5. (e) Western blot analysis of surface-purified proteins verifies splicing of the full-length receptor only when all required components were present (indicated with red arrow). Antibody targeting the C-terminus of P2X2 was used. Black arrows on the right indicate band positions of the respective constructs (Actual Mw of constructs: WT, 53 kDa; C-construct, 85 kDa; C-terminal cleavage product, C*, 46 kDa). Source data are provided as a Source Data file.

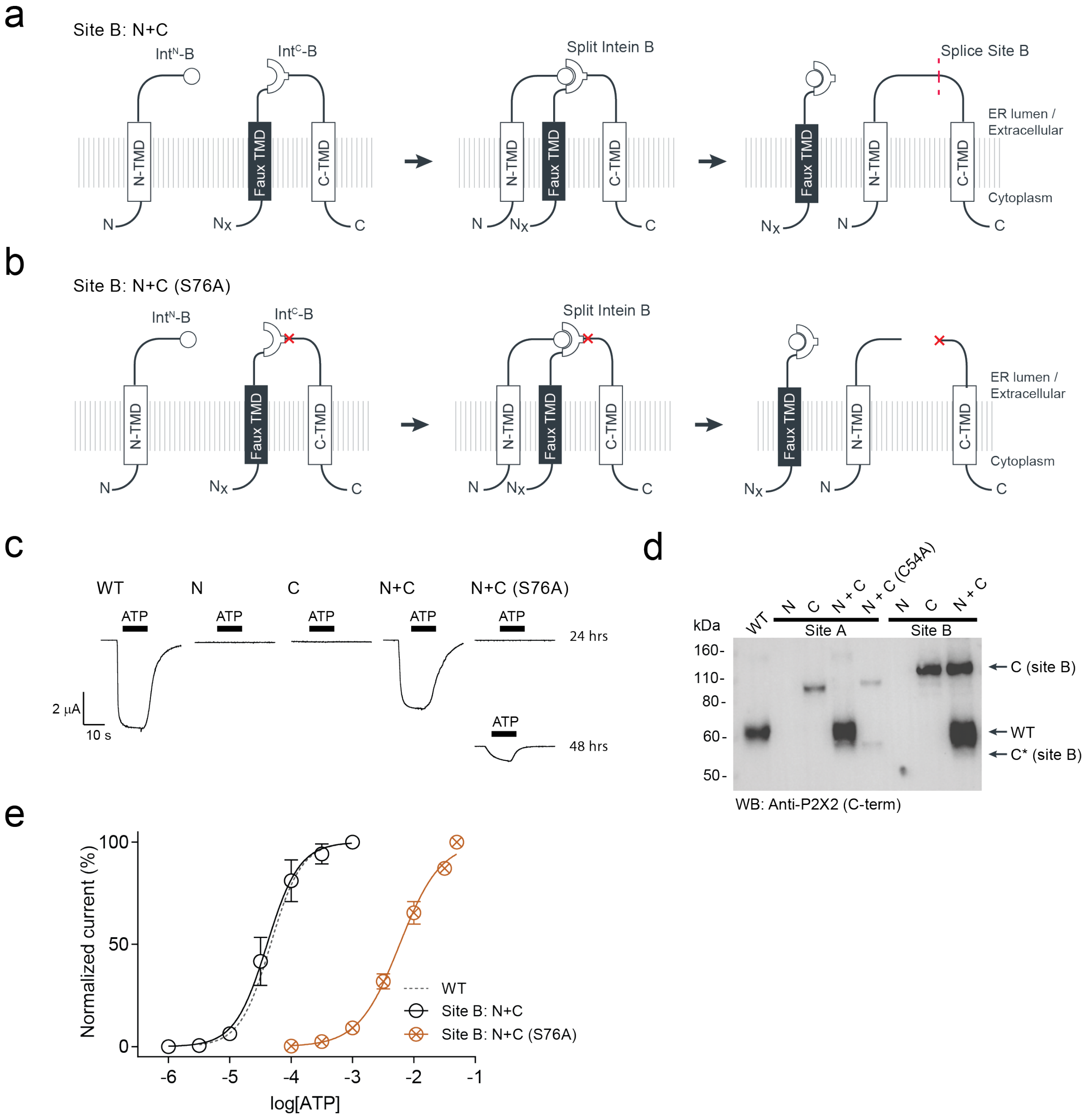

Supplementary figure 10: Single-intein PTS of the P2X2R extracellular domain at splice site B. (a) Schematic presentation of single-intein PTS at position 76 (site B) in the extracellular domain of P2X2Rs in *Xenopus laevis* oocytes. Note that a faux transmembrane helix was engineered into the C-terminal fragment to maintain its native membrane topology during protein expression. Intein B (*Ssp*DnaB^{M86}) is indicated by round symbols. (b) Schematic presentation of non-spliced P2X2 fragments expected when +1 extein residue of split intein B (position 76 of P2X2) is mutated to alanine (indicated by red cross). This prevents splicing and favors side reactions, resulting in cleavage products to accumulate (note that this will likely overrepresent the occurrence of the side reactions compared to when splicing-competent split inteins are used). (c) Representative current traces during application of 1 mM ATP (indicated by black bars) to oocytes expressing respective constructs recorded one day after injection of mRNA. ATP-induced currents were absent when N and C constructs were expressed individually. ATP-induced currents were observed only 2 days after injection of N+C (S76A) mRNA. (d) Western blot analysis of surface-purified proteins verifies splicing of the full-length receptor only when all

required components were present. Antibody targeting the C-terminus of P2X2 was used. Black arrows on the right indicate band positions of the respective site B constructs (Actual MW of constructs: WT, 53 kDa; C-construct, 97 kDa; C-terminal cleavage product, C*, 44 kDa). Note that band positions and MW of site A constructs (lanes 2-5 of the blot) are presented in Supplementary Fig 9. (e) Concentration-response curve (CRC) of reconstituted receptor indicates wild-type like functionality. Functional non-spliced construct has a significantly right-shifted CRC compared to WT. Values are displayed as mean +/- SD; WT, n = 5; N+C, n = 7; N+C(S76A), n = 4. Source data are provided as a Source Data file.

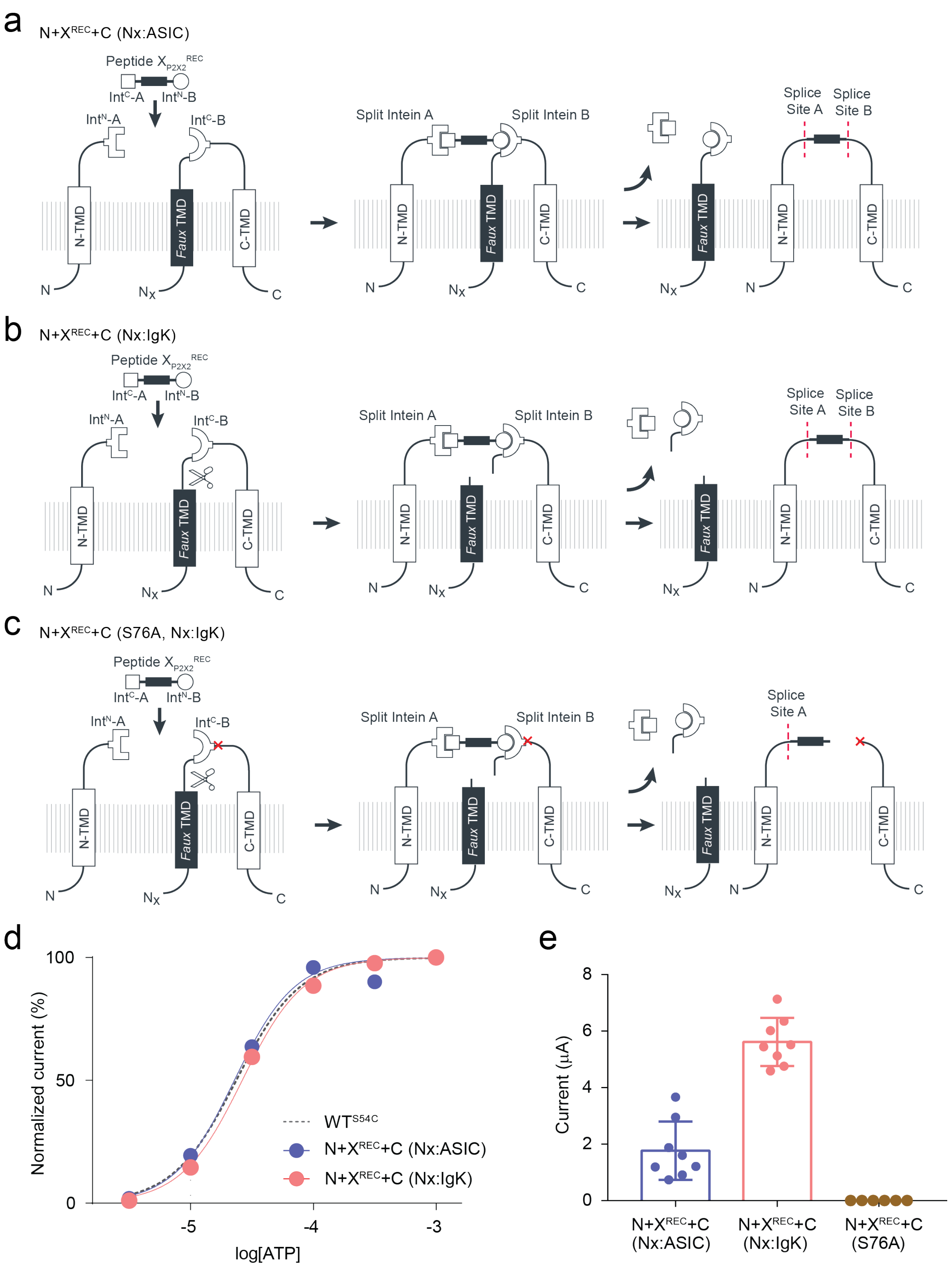
a
N+X^REC+C (Nx:ASIC)
Peptide X P2X2 REC
IntC-A
IntN-B
IntN-A
IntC-B
Split Intein A
Split Intein B
Splice Site A
Splice Site B
N-TMD
Faux TMD
C-TMD
N
Nx
C
b
N+X^REC+C (Nx:IgK)
c
N+X^REC+C (S76A, Nx:IgK)
d
Normalized current (%)
100
50
0
-5
-4
-3
log[ATP]
WT S54C
N+X^REC+C (Nx:ASIC)
N+X^REC+C (Nx:IgK)
e
Current (µA)
8
6
4
2
0
N+X^REC+C (Nx:ASIC)
N+X^REC+C (Nx:IgK)
N+X^REC+C (S76A)

Supplementary figure 11: Alternative *faux* TMDs used for C-constructs in tPTS of the P2X2R extracellular domain. (a) Schematic presentation of tPTS applied to the P2X2R extracellular domain where a non-cleavable *faux*TMD (ASIC1a N-TMD sequence, Nx:ASIC) was used for the C-construct. *Faux* transmembrane helix was engineered into the C-terminal fragment to maintain its native membrane topology during protein expression. Inteins A (*Cfa*DnaE) and B (*Ssp*DnaB^{M86}) are indicated by square and round symbols, respectively. Full-length P2X2Rs were reconstructed by recombinantly co-expressing N-/C-terminal fragments (N and C) and a recombinant peptide corresponding to amino acids 54 to 75 of the P2X2R extracellular domain (peptide X_{P2X2}^{REC}; X^{REC}) in *Xenopus laevis* oocytes. (b) Schematic of tPTS applied to the P2X2R extracellular domain where a cleavable *faux* TMD (IgK N-term signal sequence, Nx:IgK) was used for the C-construct. (c) Schematic of non-spliced P2X2 fragments expected when +1 extein residue of split intein B (position 76 of P2X2) is mutated to alanine (indicated by red cross). This prevents splicing and favors side reactions, resulting in accumulation of cleavage products (note that this will likely overrepresent the occurrence of the side reactions compared to when splicing-competent split inteins are used). (d) Concentration-response curve (CRC) of reconstituted P2X2 receptors compared to WT. Values displayed as mean +/- SD; Nx:ASIC, n = 4; Nx:IgK, n = 4). (e) Maximal current size comparison of the tPTS reconstituted receptors using the non-cleavable/cleavable *faux*TMD recorded 3 days after injection of mRNA. No current was observed for the non-spliced constructs (produced by tPTS side reactions, N+X^{REC}+C(S76A)). Values displayed as mean +/- SD; Nx:ASIC, n = 8; Nx:IgK, n = 8; S76A, n = 6. Source data are provided as a Source Data file.

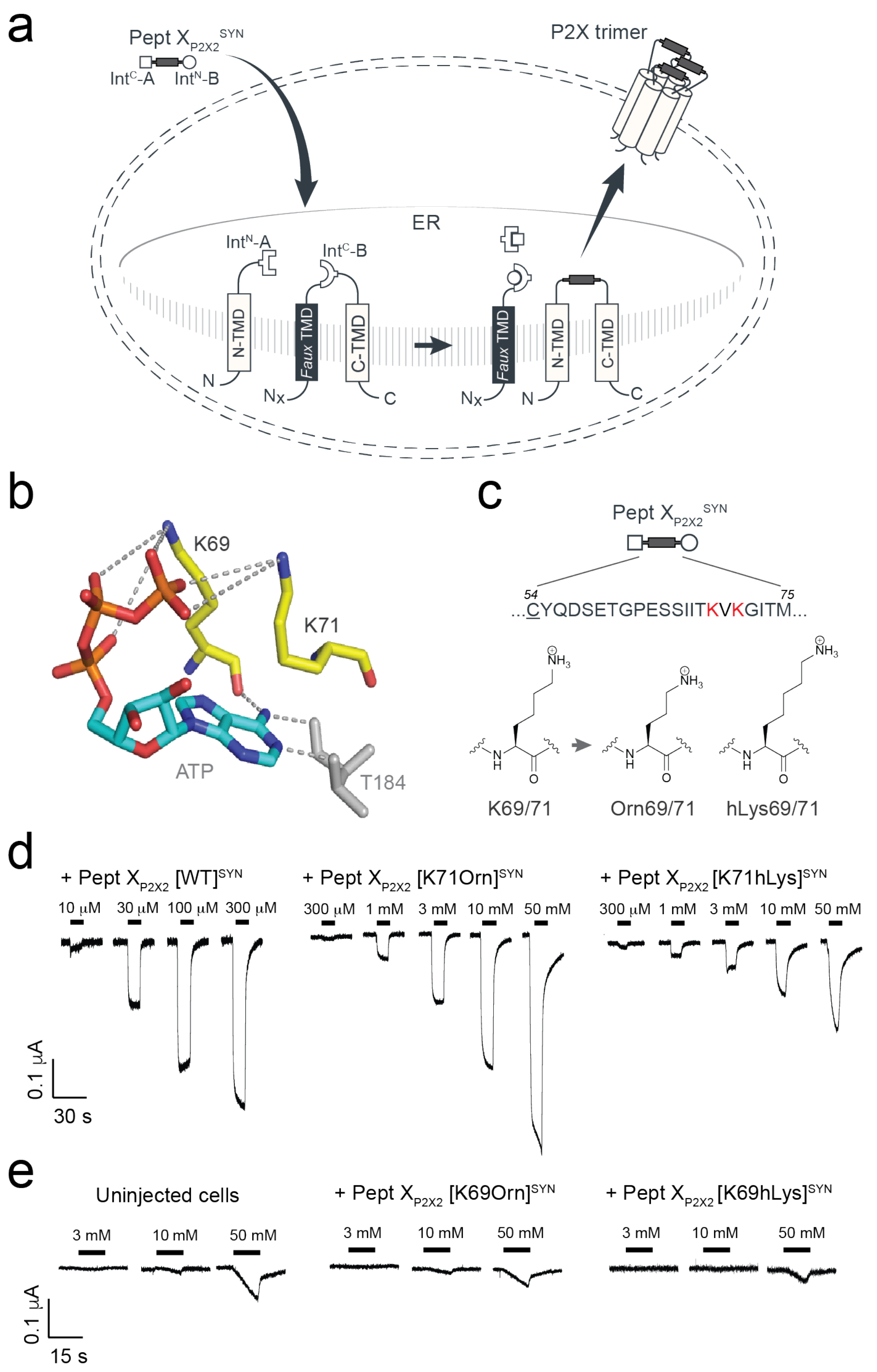

Supplementary figure 12: Incorporation of lysine derivatives at the K69 and K71 positions in the ATP binding site of P2X2 using tPTS. (a) Schematic presentation of the strategy to reconstruct full-length P2X2Rs from recombinantly expressed N-/C-terminal fragments (N and C) and a synthetic peptide (Pept $X_{P2X2}{}^{SYN}$) corresponding to amino acids 54 to 75 of the P2X2R extracellular domain in *Xenopus laevis* oocytes. Note that a *faux* transmembrane helix (*faux* TMD) was engineered into the C-terminal fragment to maintain its native membrane topology during protein expression. Inteins A (*Cfa*DnaE) and B (*Ssp*DnaB^{M86}) are indicated by square and round symbols, respectively. Peptide X was designed to include a C-terminal ER targeting KDEL signal sequence (not depicted in the schematic presentation), which is excised during the splicing process. (b) Interaction of K69 and K71 (P2X2 residue numbering) with the phosphate tail of ATP (orange) as obtained

from the crystal structure of ATP-bound hP2X3R (PDB: 5svk). (c) Sequence of peptide X_{P2X2}^{SYN} corresponding to the amino acids replaced in the P2X2 extracellular ATP binding site. Underlined residue indicates S54C mutation that was introduced to optimize the splicing reaction. Lower panel displays the chemical structures of lysine derivatives incorporated at the K69 or K71 position via chemical synthesis of peptide X_{P2X2}^{SYN}. (d) Representative ATP-induced currents when synthetic peptide with WT sequence (i.e., lysines in positions 69 and 71; left panel) or synthetic peptide X variants with K71 modifications (K71Orn or K71hLys) was incorporated using tPTS. (e) Representative ATP-induced currents when synthetic peptide X variants with K69 modifications (K69Orn or K69hLys) was incorporated using tPTS. Small ATP-induced currents observed only at very high ATP concentrations (>10 mM) were similar to those observed in uninjected cells (left panel).

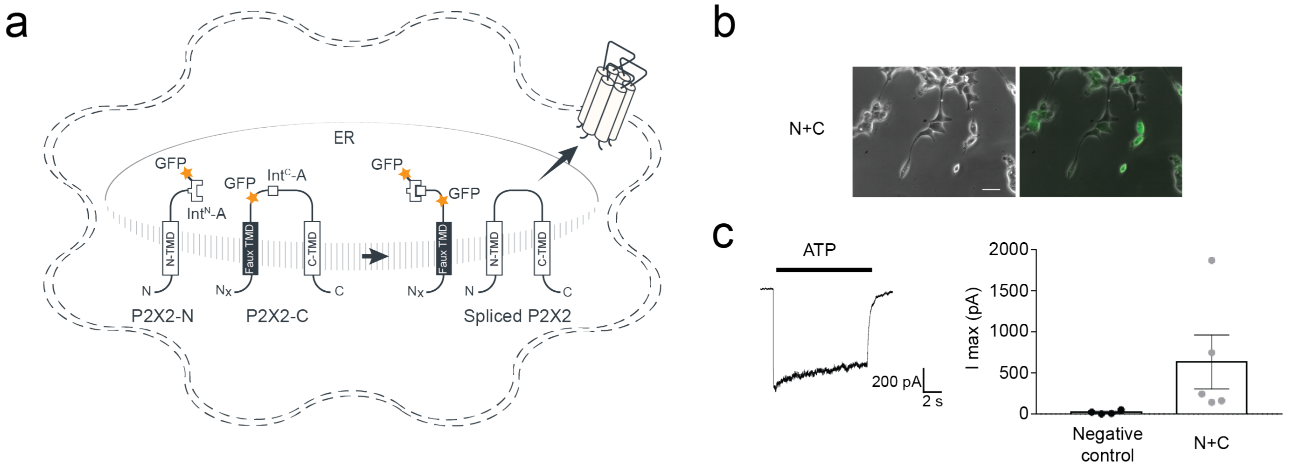

Supplementary figure 13: PTS of the P2X2R extracellular domain in HEK293 cells. (a) Schematic of constructs used for single PTS of P2X2Rs in HEK293 cells. Note that a faux transmembrane helix was engineered into the C-terminal fragment to maintain its native membrane topology during protein expression. Intein A (*Cfa*DnaE) is indicated by square symbols and GFP is indicated by an orange star. (b) GFP-fused P2X2-split intein constructs were successfully transfected into HEK293 cells. Scale bar: 50 µm. (c) ATP-induced current recorded during application of 1 mM ATP in HEK293 cells transfected with both P2X2 N and C constructs. Cells transfected with GFP only were used as a negative control. Values are displayed as mean +/- SD; Negative control, n = 4; N+C, n = 5.

Supplementary Table 1: Activation and steady-state inactivation parameters of $Na_V1.5$ constructs

Construct	Activation V_{50} (mV)	*n*	Inactivation V_{50} (mV)	*n*
WT	-35.5 ± 1.6	12	-73.1 ± 2.2	12
DIII-DIV linker site				
WT^{N1472C}	-34.1 ± 1.8	9	-68.9 ± 3.0	16
$[WT]^{Rec}$	-34.3 ± 4.2	13	-68.7 ± 4.8	14
$[WT^{NM}]^{Syn}$	-36.2 ± 2.3	10	-70.6 ± 2.4	10
$[tAcK1479]^{Syn}$	-35.3 ± 1.9	17	-67.6 ± 2.8	21
$[phY1495]^{Syn}$	-36.0 ± 2.8	12	-56.4 ± 2.5	20
$[tAcK1479+phY1495]^{Syn}$	-35.0 ± 1.7	14	-55.7 ± 1.4	14
DI-DII linker site				
$WT^{A505C,M506F}$	-32.8 ± 3.3	17	-72.2 ± 3.2	17
$[WT]^{Rec}$	-33.5 ± 4.1	14	-75.5 ± 2.6	13
$[WT^{NM}]^{Syn}$	-34.0 ± 2.3	7	-78.2 ± 1.7	8
$[meR513]^{Syn}$	-34.0 ± 3.6	15	-75.1 ± 3.3	15
$[phS516]^{Syn}$	-33.7 ± 2.0	19	-74.8 ± 2.4	21
$[meR513+phS516]^{Syn}$	-33.2 ± 3.0	19	-75.6 ± 2.8	21

Supplementary Table 2: Primers used for PCR mutagenesis

Mutation	Oligo Name	*Sequence (5′–3′)*
Nav1.5 DIII-DIV linker site		
WT: N1472C	Nav1.5wt_N1472C_fwd	GTGATCATCGACTGCTTCAACCAGCAGAAG
	Nav1.5wt_N1472C_rev	CTTCTGCTGGTTGAAGCAGTCGATGATCAC
$X_{Nav1.5}^{REC}$: C1472A	Nav1.5x_C1472A_fwd	GCCTGGTGGCCAGCAACGCCTTCAACCAGCAGAAGAAG
	Nav1.5x_C1472A_rev	CTTCTTCTGCTGGTTGAAGGCGTTGCTGGCCACCAGGC
C construct: S1503A	Nav1.5c_S1503A_fwd	GACATCATCGTGCACAACGCCAAGAAGCCCCAGAAGCCT
	Nav1.5c_S1503A_rev	AGGCTTCTGGGGCTTCTTGGCGTTGTGCACGATGATGTC
Nav1.5 DI-DII linker site		
WT: A505C,M506F	Nav1.5wt_A505C_fwd	GATGGCCCCAGATGCTTCAACCACCTGTCTCTG
	Nav1.5wt_A505C_rev	CAGAGACAGGTGGTTGAAGCATCTGGGGCCATC
$X_{Nav1.5}^{REC}$: C505A	Nav1.5x_C505A_fwd	GCCTGGTGGCCAGCAACGCCTTCAACCACCTGAGCCTG
	Nav1.5x_C505A_rev	CAGGCTCAGGTGGTTGAAGGCGTTGCTGGCCACCAGGC
C construct: S528A	Nav1.5c_S528A_fwd	GACATCATCGTGCACAACGCCATCTTCACCTTCCGGCGG
	Nav1.5c_S528A_rev	CCGCCGGAAGGTGAAGATGGCGTTGTGCACGATGATGTC
eGFP		
X_{GFP}^{REC}: C65A	GFPx_C65A_fwd	CTGGTGGCCAGCAACGCTTACGGCGTGCAGTGCTTC
	GFPx_C65A_rev	GAAGCACTGCACGCCGTAAGCGTTGCTGGCCACCAG
C construct: S86A	GFPc_S86A_fwd	CAACGACATCATCGTGCACAACGCCGCCATGCCTGAG
	GFPc_S86A_rev	ACGTAGCCCTCAGGCATGGCGGCGTTGTGCACGATG
P2X2		
WT: S54C	P2X2wt_S54C_fwd	GTTCATCGTGCAGAAGTGCTACCAGGACAG
	P2X2wt_S54C_rev	CTGTCCTGGTAGCACTTCTGCACGATGAAC
C construct (site A): C54A	P2X2c_C54A_fwd	CCAGCAACGCCTACCAGGATTCTGAGAC
	P2X2c_C54A_rev	GTCTCAGAATCCTGGTAGGCGTTGCTGG
C construct (site B): S76A	P2X2c_S76A_fwd	GACATCATCGTGCACAACGCCGAGGACAAAGTGTG
	P2X2c_S76A_rev	CACACTTTGTCCTCGGCGTTGTGCACGATGATGTC

Supplementary methods

Design of plasmid DNA constructs

Plasmid DNA constructs were designed to encode for the respective protein sequences shown below:

hNav1.5 DI-DII linker splicing constructs

N-construct

pUNIV - hNav1.5(aa 1-504) - **CfaDnaE**$_{\mathbf{N101}}$ - *HA tag linker* - *ER retention signal*

MANFLLPRGTSSFRRFTRESLAAIEKRMAEKQARGSTTLQESREGLPEEEAPRPQLDLQA
SKKLPDLYGNPPQELIGEPLEDLDPFYSTQKTFIVLNKGKTIFRFSATNALYVLSPFHPIRR
AAVKILVHSLFNMLIMCTILTNCVFMAQHDPPPWTKYVEYTFTAIYTFESLVKILARGFCLH
AFTFLRDPWNWLDFSVIIMAYTTEFVDLGNVSALRTFRVLRALKTISVISGLKTIVGALIQSV
KKLADVMVLTVFCLSVFALIGLQLFMGNLRHKCVRNFTALNGTNGSVEADGLVWESLDLY
LSDPENYLLKNGTSDVLLCGNSSDAGTCPEGYRCLKAGENPDHGYTSFDSFAWAFLALF
RLMTQDCWERLYQQTLRSAGKIYMIFFMLVIFLGSFYLVNLILAVVAMAYEEQNQATIAET
EEKEKRFQEAMEMLKKEHEALTIRGVDTVSRSSLEMSPLAPVNSHERRSKRRKRMSSGT
EECGEDRLPKSDSEDGPR**CLSYDTEILTVEYGFLPIGKIVEERIECTVYTVDKNGFVYTQP**
IAQWHNRGEQEVFEYCLEDGSIIRATKDHKFMTTDGQMLPIDEIFERGLDLKQVDGLP*YP*
*YDVPDYAYPYDVPDYLLDALTLASSRGPLRKRSVAVAKAKPKFSISPDSLS*PRKKFQ*

X-construct ‘Rec’

pUNIV - **CfaDnaE**$_{\mathbf{C35}}$ - hNav1.5(aa 505-527, A505C, M506F) - **SspDnaB**$^{\mathbf{M86}}_{\mathbf{N11}}$

VKIISRKSLGTQNVYDIGVEKDHNFLLKNGLVASNCFNHLSLTRGLSRTSMKPRSSRG**CI**
SGDSLISLA

C-construct

pUNIV – *ER retention signal* - *linker* - **SspDnaB**$^{\mathbf{M86}}_{\mathbf{C143}}$ - hNav1.5(aa 528-2016)

*MLLDALTLASSRGPLRKRSVAVAKAKPKFSISPDSLSGSAGSAAGSGEF***STGKRVPIKDL**
LGEKDFEIWAINEQTMKLESAKVSRVFCTGKKLVYTLKTRLGRTIKATANHRFLTIDGWK
RLDELSLKEHIALPRKLESSSLQLAPEIEKLPQSDIYWDPIVSITETGVEEVFDLTVPGLRN
FVANDIIVHNSIFTFRRRDLG**S**EADFADDENSTAGESESHHTSLLVPWPLRRTSAQGQPS
PGTSAPGHALHGKKNSTVDCNGVVSLLGAGDPEATSPGSHLLRPVMLEHPPDTTTPSEE
PGGPQMLTSQAPCVDGFEEPGARQRALSAVSVLTSALEELEESRHKCPPCWNRLAQRY
LIWECCPLWMSIKQGVKLVVMDPFTDLTITMCIVLNTLFMALEHYNMTSEFEEMLQVGNL
VFTGIFTAEMTFKIIALDPYYYFQQGWNIFDSIIVILSLMELGLSRMSNLSVLRSFRLLRVFKL
AKSWPTLNTLIKIIGNSVGALGNLTLVLAIIVFIFAVVGMQLFGKNYSELRDSDSGLLPRWH
MMDFFHAFLIIFRILCGEWIETMWDCMEVSGQSLCLLVFLLVMVIGNLVVLNLFLALLLSSF

SADNLTAPDEDREMNNLQLALARIQRGLRFVKRTTWDFCCGLLRQRPQKPAALAAQGQL
PSCIATPYSPPPPETEKVPPTRKETRFEEGEQPGQGTPGDPEPVCVPIAVAESDTDDQEE
DEENSLGTEEESSKQQESQPVSGGPEAPPDSRTWSQVSATASSEAEASASQADWRQQ
WKAEPQAPGCGETPEDSCSEGSTADMTNTAELLEQIPDLGQDVKDPEDCFTEGCVRRC
PCCAVDTTQAPGKVWWRLRKTCYHIVEHSWFETFIIFMILLSSGALAFEDIYLEERKTIKVL
LEYADKMFTYVFVLEMLLKWVAYGFKKYFTNAWCWLDFLIVDVSLVSLVANTLGFAEMGP
IKSLRTLRALRPLRALSRFEGMRVVVNALVGAIPSIMNVLLVCLIFWLIFSIMGVNLFAGKFG
RCINQTEGDLPLNYTIVNNKSQCESLNLTGELYWTKVKVNFDNVGAGYLALLQVATFKGW
MDIMYAAVDSRGYEEQPQWEYNLYMYIYFVIFIIFGSFFTLNLFIGVIIDNFNQQKKKLGGQ
DIFMTEEQKKYYNAMKKLGSKKPQKPIPRPLNKYQGFIFDIVTKQAFDVTIMFLICLNMVTM
MVETDDQSPEKINILAKINLLFVAIFTGECIVKLAALRHYYFTNSWNIFDFVVVILSIVGTVLS
DIIQKYFFSPTLFRVIRLARIGRILRLIRGAKGIRTLLFALMMSLPALFNIGLLLFLVMFIYSIFG
MANFAYVKWEAGIDDMFNFQTFANSMLCLFQITTSAGWDGLLSPILNTGPPYCDPTLPNS
NGSRGDCGSPAVGILFFTTYIIISFLIVVNMYIAIILENFSVATEESTEPLSEDDFDMFYEIWE
KFDPEATQFIEYSVLSDFADALSEPLRIAKPNQISLINMDLPMVSGDRIHCMDILFAFTKRVL
GESGEMDALKIQMEEKFMAANPSKISYEPITTTLRRKHEEVSAMVIQRAFRRHLLQRSLKH
ASFLFRQQAGSGLSEEDAPEREGLIAYVMSENFSRPLGPPSSSSISSTSFPPSYDSVTRA
TSDNLQVRGSDYSHSEDLADFPPSPDRDRESIV*

<u>Nav1.5 DIII-DIV linker splicing constructs</u>

N-construct

pUNIV - hNav1.5(aa 1-1471) - **CfaDnaE$_{N101}$** – *HA tag linker* - <u>ER retention signal</u>

MANFLLPRGTSSFRRFTRESLAAIEKRMAEKQARGSTTLQESREGLPEEEAPRPQLDLQA
SKKLPDLYGNPPQELIGEPLEDLDPFYSTQKTFIVLNKGKTIFRFSATNALYVLSPFHPIRR
AAVKILVHSLFNMLIMCTILTNCVFMAQHDPPPWTKYVEYTFTAIYTFESLVKILARGFCLH
AFTFLRDPWNWLDFSVIIMAYTTEFVDLGNVSALRTFRVLRALKTISVISGLKTIVGALIQSV
KKLADVMVLTVFCLSVFALIGLQLFMGNLRHKCVRNFTALNGTNGSVEADGLVWESLDLY
LSDPENYLLKNGTSDVLLCGNSSDAGTCPEGYRCLKAGENPDHGYTSFDSFAWAFLALF
RLMTQDCWERLYQQTLRSAGKIYMIFFMLVIFLGSFYLVNLILAVVAMAYEEQNQATIAET
EEKEKRFQEAMEMLKKEHEALTIRGVDTVSRSSLEMSPLAPVNSHERRSKRRKRMSSGT
EECGEDRLPKSDSEDGPRAMNHLSLTRGLSRTSMKPRSSRGSIFTFRRRDLGSEADFAD
DENSTAGESESHHTSLLVPWPLRRTSAQGQPSPGTSAPGHALHGKKNSTVDCNGVVSL
LGAGDPEATSPGSHLLRPVMLEHPPDTTTPSEEPGGPQMLTSQAPCVDGFEEPGARQR
ALSAVSVLTSALEELEESRHKCPPCWNRLAQRYLIWECCPLWMSIKQGVKLVVMDPFTD
LTITMCIVLNTLFMALEHYNMTSEFEEMLQVGNLVFTGIFTAEMTFKIIALDPYYYFQQGWN
IFDSIIVILSLMELGLSRMSNLSVLRSFRLLRVFKLAKSWPTLNTLIKIIGNSVGALGNLTLVL
AIIVFIFAVVGMQLFGKNYSELRDSDSGLLPRWHMMDFFHAFLIIFRILCGEWIETMWDCM
EVSGQSLCLLVFLLVMVIGNLVVLNLFLALLLSSFSADNLTAPDEDREMNNLQLALARIQR
GLRFVKRTTWDFCCGLLRQRPQKPAALAAQGQLPSCIATPYSPPPPETEKVPPTRKETR
FEEGEQPGQGTPGDPEPVCVPIAVAESDTDDQEEDEENSLGTEEESSKQQESQPVSGG
PEAPPDSRTWSQVSATASSEAEASASQADWRQQWKAEPQAPGCGETPEDSCSEGSTA
DMTNTAELLEQIPDLGQDVKDPEDCFTEGCVRRCPCCAVDTTQAPGKVWWRLRKTCYHI
VEHSWFETFIIFMILLSSGALAFEDIYLEERKTIKVLLEYADKMFTYVFVLEMLLKWVAYGFK
KYFTNAWCWLDFLIVDVSLVSLVANTLGFAEMGPIKSLRTLRALRPLRALSRFEGMRVVV
NALVGAIPSIMNVLLVCLIFWLIFSIMGVNLFAGKFGRCINQTEGDLPLNYTIVNNKSQCESL

NLTGELYWTKVKVNFDNVGAGYLALLQVATFKGWMDIMYAAVDSRGYEEQPQWEYNLY
MYIYFVIFIIFGSFFTLNLFIGVIID**CLSYDTEILTVEYGFLPIGKIVEERIECTVYTVDKNGFVY**
TQPIAQWHNRGEQEVFEYCLEDGSIIRATKDHKFMTTDGQMLPIDEIFERGLDLKQVDGL
P*YPYDVPDYAYPYDVPDYLLDALTLASSRGPLRKRSVAVAKAKPKFSISPDSLS*PRKKFQ*

X-construct ‘Rec’

pUNIV - **CfaDnaE**$_{C35}$ - hNav1.5(aa 1472-1502, N1472C) - **SspDnaB**$^{M86}_{N11}$

VKIISRKSLGTQNVYDIGVEKDHNFLLKNGLVASNCFNQQKKKLGGQDIFMTEEQKKYYN
AMKKLG**CISGDSLISLA**

C-construct

pUNIV – *ER retention signal* - *linker* - **SspDnaB**$^{M86}_{C143}$ - hNav1.5(aa 1503-2016)

*MLLDALTLASSRGPLRKRSVAVAKAKPKFSISPDSLSGSAGSAAGSGEF***STGKRVPIKDL**
LGEKDFEIWAINEQTMKLESAKVSRVFCTGKKLVYTLKTRLGRTIKATANHRFLTIDGWK
RLDELSLKEHIALPRKLESSSLQLAPEIEKLPQSDIYWDPIVSITETGVEEVFDLTVPGLRN
FVANDIIVHNSKKPQKPIPRPLNKYQGFIFDIVTKQAFDVTIMFLICLNMVTMMVETDDQSP
EKINILAKINLLFVAIFTGECIVKLAALRHYYFTNSWNIFDFVVVILSIVGTVLSDIIQKYFFSPT
LFRVIRLARIGRILRLIRGAKGIRTLLFALMMSLPALFNIGLLLFLVMFIYSIFGMANFAYVKW
EAGIDDMFNFQTFANSMLCLFQITTSAGWDGLLSPILNTGPPYCDPTLPNSNGSRGDCGS
PAVGILFFTTYIIISFLIVVNMYIAIILENFSVATEESTEPLSEDDFDMFYEIWEKFDPEATQFIE
YSVLSDFADALSEPLRIAKPNQISLINMDLPMVSGDRIHCMDILFAFTKRVLGESGEMDALK
IQMEEKFMAANPSKISYEPITTTLRRKHEEVSAMVIQRAFRRHLLQRSLKHASFLFRQQAG
SGLSEEDAPEREGLIAYVMSENFSRPLGPPSSSSISSTSFPPSYDSVTRATSDNLQVRGS
DYSHSEDLADFPPSPDRDRESIV*

rP2X2 extracellular site splicing constructs

Single split intein A (CfaDnaE) splicing constructs

N-construct

pUNIV - rP2X2(aa 1-53) - ***CfaDnaE***$_{N101}$ – *linker* - SEP

MVRRLARGCWSAFWDYETPKVIVVRNRRLGFVHRMVQLLILLYFVWYVFIVQK**CLSYDTE
ILTVEYGFLPIGKIVEERIECTVYTVDKNGFVYTQPIAQWHNRGEQEVFEYCLEDGSIIRAT
KDHKFMTTDGQMLPIDEIFERGLDLKQVDGLP**_GSAGSAAGSGEF_SKGEELFTGVVPILVE
LDGDVNGHKFSVSGEGEGDATYGKLTLKFICTTGKLPVPWPTLVTTLTYGVQCFSRYPD
HMKRHDFFKSAMPEGYVQERTIFFKDDGNYKTRAEVKFEGDTLVNRIELKGIDFKEDGNIL
GHKLEYNYNDHQVYIMADKQKNGIKANFKIRHNIEDGGVQLADHYQQNTPIGDGPVLLPD
NHYLFTTSTLSKDPNEKRDHMVLLEFVTAAGITHGMDELYK*

C-construct

pUNIV - Nx(rP2X2 aa 1-53) - *linker* - SEP - *linker* - **CfaDnaE**$_{C35}$ - rP2X2(aa 54-472, S54C)

MVRRLARGCWSAFWDYETPKVIVVRNRRLGFVHRMVQLLILLYFVWYVFIVQK_GSAGSA
AGSGEF_SKGEELFTGVVPILVELDGDVNGHKFSVSGEGEGDATYGKLTLKFICTTGKLPV
PWPTLVTTLTYGVQCFSRYPDHMKRHDFFKSAMPEGYVQERTIFFKDDGNYKTRAEVKF
EGDTLVNRIELKGIDFKEDGNILGHKLEYNYNDHQVYIMADKQKNGIKANFKIRHNIEDGG
VQLADHYQQNTPIGDGPVLLPDNHYLFTTSTLSKDPNEKRDHMVLLEFVTAAGITHGMDE
LYK_GSAGSAAGSGEF_**VKIISRKSLGTQNVYDIGVEKDHNFLLKNGLVASN**CYQDSETGP
ESSIITKVKGITMSEDKVWDVEEYVKPPEGGSVVSIITRIEVTPSQTLGTCPESMRVHSSTC
HSDDDCIAGQLDMQGNGIRTGHCVPYYHGDSKTCEVSAWCPVEDGTSDNHFLGKMAPN
FTILIKNSIHYPKFKFSKGNIASQKSDYLKHCTFDQDSDPYCPIFRLGFIVEKAGENFTELAH
KGGVIGVIINWNCDLDLSESECNPKYSFRRLDPKYDPASSGYNFRFAKYYKINGTTTTRTL
IKAYGIRIDVIVHGQAGKFSLIPTIINLATALTSIGVGSFLCDWILLTFMNKNKLYSHKKFDKV
RTPKHPSSRWPVTLALVLGQIPPPPSHYSQDQPPSPPSGEGPTLGEGAELPLAVQSPRP
CSISALTEQVVDTLGQHMGQRPPVPEPSQQDSTSTDPKGLAQL*

Double split intein splicing constructs

X-construct 'Rec'

pUNIV - **CfaDnaE**$_{C35}$ - rP2X2(aa 54-75, S54C) - **SspDnaB**$^{M86}_{N11}$ – *linker* – *ER targeting signal*

VKIISRKSLGTQNVYDIGVEKDHNFLLKNGLVASNCYQDSETGPESSIITKVKGITM**CISGD
SLISLA**_SSGESKDEL_*

C-construct

pUNIV - Nx(IgK cleavable) – *HA tag linker* - **SspDnaB**$^{M86}_{C143}$ - rP2X2(aa 76-472) - *myc tag*

METDTLLLWVLLLWVPGSTG^D*YPYDVPDYAGSAGSAAGSGEF***STGKRVPIKDLLGEKD**
FEIWAINEQTMKLESAKVSRVFCTGKKLVYTLKTRLGRTIKATANHRFLTIDGWKRLDEL
SLKEHIALPRKLESSSLQLAPEIEKLPQSDIYWDPIVSITETGVEEVFDLTVPGLRNFVAN
DIIVHNSEDKVWDVEEYVKPPEGGSVVSIITRIEVTPSQTLGTCPESMRVHSSTCHSDDDC
IAGQLDMQGNGIRTGHCVPYYHGDSKTCEVSAWCPVEDGTSDNHFLGKMAPNFTILIKN
SIHYPKFKFSKGNIASQKSDYLKHCTFDQDSDPYCPIFRLGFIVEKAGENFTELAHKGGVI
GVIINWNCDLDLSESECNPKYSFRRLDPKYDPASSGYNFRFAKYYKINGTTTTRTLIKAYGI
RIDVIVHGQAGKFSLIPTIINLATALTSIGVGSFLCDWILLTFMNKNKLYSHKKFDKVRTPKH
PSSRWPVTLALVLGQIPPPPSHYSQDQPPSPPSGEGPTLGEGAELPLAVQSPRPCSISAL
TEQVVDTLGQHMGQRPPVPEPSQQDSTSTDPKGLAQL*EQKLISEEDL**

eGFP splicing constructs

N-construct

Pcdna3.1 - eGFP(aa 1-64)**- CfaDnaE$_{N101}$**

MVSKGEELFTGVVPILVELDGDVNGHKFSVSGEGEGDATYGKLTLKFICTTGKLPVPWPT
LVTTL**CLSYDTEILTVEYGFLPIGKIVEERIECTVYTVDKNGFVYTQPIAQWHNRGEQEVF**
EYCLEDGSIIRATKDHKFMTTDGQMLPIDEIFERGLDLKQVDGLP*

X-construct

Pcdna3.1 – *TAT cpp* – *linker* **– CfaDnaE$_{C35}$** - eGFP (aa 65-85, T65C) **- SspDnaB$^{M86}_{N11}$**

M*GRKKRRQRRRPQGSAGSAAGSGEF***VKIISRKSLGTQNVYDIGVEKDHNFLLKNGLVA**
SNCYGVQCFSRYPDHMKQHDFFK**CISGDSLISLA***

C-construct

Pcdna3.1 **–** HA tag **–** *linker* **- SspDnaB$^{M86}_{C143}$** - eGFP (aa 86-238)

MYPYDVPDYA*GSAGSAAGSGEF***STGKRVPIKDLLGEKDFEIWAINEQTMKLESAKVSRV**
FCTGKKLVYTLKTRLGRTIKATANHRFLTIDGWKRLDELSLKEHIALPRKLESSSLQLAP
EIEKLPQSDIYWDPIVSITETGVEEVFDLTVPGLRNFVANDIIVHNSAMPEGYVQERTIFFK
DDGNYKTRAEVKFEGDTLVNRIELKGIDFKEDGNILGHKLEYNYNSHNVYIMADKQKNGIK
VNFKIRHNIEDGSVQLADHYQQNTPIGDGPVLLPDNHYLSTQSALSKDPNEKRDHMVLLE
FVTAAGITLGMDELYK*

Peptide synthesis and purification

General

All reagents and solvents were of analytical grade and used without further purification as obtained from commercial suppliers (Iris, Combi-Blocks, Rapp Polymere, Fluoro Chem, Sigma Aldrich). Anhydrous solvents were purchased from Sigma Aldrich. Reactions were conducted under an atmosphere of nitrogen whenever anhydrous solvents were used. Evaporation of solvents was carried out under reduced pressure at temperatures below 45

°C. Loading of resin during solid phase peptide synthesis was checked spectrophotometrically, quantifying the amount of Fmoc released upon cleavage of a small sample[2].

Low resolution mass spectra were recorded on a MALDI-TOF Bruker Microflex LT/SH system, and samples were prepared using SA (sinapic acid) matrix dissolved in water–MeCN–TFA (50:50:0.1, v/v/v). The calculated mass reported is the most intense peak (100% relative intensity), predicted with mMass software.

High resolution mass spectra (HR-MS) were recorded on a SOLARIX ESI MALDI from Bruker Daltronik. Samples were dissolved in MeCN–water–FA (50:50:0.1, v/v/v) and were analyzed by ESI. The calculated mass reported is the most intense peak predicted with mMass software (100% relative intensity) in the isotopes pattern, which is compared to the most intense peak experimentally found in the isotopes pattern.

Unless otherwise stated, the amino acids used for solid phase peptide synthesis were:

Fmoc-Ala-OH; Fmoc-Cys(Trt)-OH; Fmoc-Phe-OH; Fmoc-Gly-OH; Fmoc-Ile-OH; Fmoc-Lys(Boc)-OH; Fmoc-Leu-OH; Fmoc-Pro-OH; Fmoc-His(Trt)-OH; Fmoc-Asn(Trt)-OH; Fmoc-Gln(Trt)-OH; Fmoc-Arg(Pbf)-OH; Fmoc-Ser(^{t}Bu)-OH; Fmoc-Thr(^{t}Bu)-OH; Fmoc-Tyr(^{t}Bu)-OH; Fmoc-Asp(^{t}Bu)-OH; Fmoc-Glu(^{t}Bu)-OH; Fmoc-Met-OH; Fmoc-Val-OH.

Fmoc-tAcLys-OH was kindly donated by prof. Christian A. Olsen (University of Copenhagen).

Chemical ligations of peptide fragments were monitored by diluting 2.5 µL of ligation mixture in water–MeCN (8:2, v/v, 100 µL). The obtained solution was checked by MALDI-TOF and analytical HPLC. Illustrative chromatograms (λ 210 nm) and MALDI-TOF spectra are shown for every ligation.

Analytical and preparative chromatography

Analytical reversed-phase HPLC was performed on an Agilent 1100 LC system equipped with a C8 Phenomenex Kinetex column [250 mm × 4.60, 5 µm, 100 Å] and a diode array UV detector, using a gradient and rising eluent II (0.1% TFA in MeCN) in eluent I (water–MeCN–TFA, 95:5:0.1, v/v/v) linearly from 0% to 40% over 40 min, with a flow rate of 1.2 mL/min at 40 °C.

Preparative reversed-phase HPLC was performed on an Agilent 1260 Infinity system equipped with a C18 Phenomenex Luna column [250 mm × 21.2 mm, 5 µm, 100 Å] or a C8 Phenomenex Luna column [250 mm × 21.2 mm, 5 µm, 100 Å] and a diode array UV detector, using a gradient of eluent I (water–MeCN–TFA, 95:5:0.1, v/v/v) and eluent II (0.1% TFA in MeCN) as specified for each compound, with a flow rate of 20 mL/min.

Loading of 2-chlorotrityl chloride polystyrene resin

2-Chlorotrityl chloride resin (250 mg, 0.35 mmol) was transferred in a polypropylene syringe equipped with a fritted disk and swollen in anhydrous CH_2Cl_2 for 45 min, followed by washing with anhydrous CH_2Cl_2 (2×). *i*-Pr_2NEt (61 µL, 0.35 mmol, 1.0 equiv) was added to a suspension of Fmoc-Ala-OH (44 mg, 0.14 mmol, 0.4 equiv) in anhydrous CH_2Cl_2 (1.5 mL) and the obtained solution was added to the resin. The suspension was agitated for 90 min, after which it was washed with DMF (4×) and CH_2Cl_2 (4×). After loading determination, the unreacted sites on resin were capped by incubating the resin with a mixture of CH_2Cl_2–MeOH–*i*-Pr_2NEt (1.7:0.25:0.12, v/v/v, 2.1 mL) for 60 min, followed by washings with CH_2Cl_2 (4×).

Fmoc-3-amino-4-(methylamino)benzoic acid (Fmoc-MeDbz-OH)

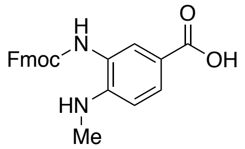

Fmoc-MeDbz-OH was synthesized essentially as previously described[3–5].

Briefly, 4-fluoro-3-nitrobenzoic acid was dissolved in methanol, followed by $MeNH_2$ (40% solution in water, 10 equiv). The reaction mixture turned bright orange and was stirred at room temperature for 20 h, after which the reaction was poured into water. The obtained solution was cooled with an ice bath and acidified with conc. HCl. The resulting bright yellow precipitate was isolated by filtration, washed with cold water and then dried under high vacuum overnight to give 4-methylamine-3-nitrobenzoic acid as a yellow solid.

4-Methylamine-3-nitrobenzoic acid obtained in the previous step was hydrogenated over Pd/C (10% wt) in methanol at atmospheric pressure and at room temperature. The reaction mixture was stirred overnight, during which it turned black. The catalyst was removed by filtration through Celite® and the clear, black filtrate was evaporated under reduced pressure to obtain 3-amino-4-(methylamino)benzoic acid as a black solid.

3-Amino-4-(methylamino)benzoic acid obtained in the previous step was suspended in a mixture of MeCN–water (1:1, v/v). Upon addition of *i*-Pr_2NEt (0.95 equiv) the reaction mixture turned into a black solution. Fmoc chloride (0.90 equiv) was dissolved in MeCN and was added dropwise to the reaction mixture at room temperature. Upon complete addition of Fmoc chloride, the reaction mixture was stirred for further 45 min, after which the MeCN was evaporated under reduced pressure. The obtained slurry was filtered and the isolated solid was washed several times with cold water and cold MeCN. Drying overnight under high vacuum gave Fmoc-3-amino-4-(methylamino)benzoic acid (Fmoc-MeDbz-OH) as a grey solid.

Fmoc-MeDbz-Gly PHB TentaGel resin

PHB TentaGel resin (2.0 g, 0.4 mmol) was transferred in a polypropylene syringe equipped with a fritted disk and swollen in CH_2Cl_2 for 30 min, followed by washing with anhydrous CH_2Cl_2 (2×). In parallel, *N*-methylimidazole (120 µL, 1.5 mmol, 3.75 equiv) was added to a solution of Fmoc-Gly-OH (595 mg, 2 mmol, 5.0 equiv) in anhydrous DMF–CH_2Cl_2 (7:1, v/v, 8 mL), followed by MSNT (593 mg, 2 mmol, 5.0 equiv). The obtained solution was added to the resin and the suspension was agitated at room temperature for two hours. The resin was then washed with DMF (3×) and CH_2Cl_2 (3×). The loading procedure was repeated once. The unreacted sites were capped *via* treatment with a solution of acetic anhydride (151 µL, 1.6 mmol, 4.0 equiv to original resin loading) and *i*-Pr_2NEt (418 µL, 2.4 mmol, 6.0 equiv to original resin loading) in CH_2Cl_2 (8 mL) for one hour. The resin was then washed with CH_2Cl_2 (5×) and Fmoc-deprotected *via* treatment with piperidine–DMF (1:4, v/v, 12 mL) for 2 min, followed by a second treatment for 15 min, after which the resin was washed with DMF (5×).

Fmoc-MeDbz-OH (388 mg, 1.0 mmol, 2.5 equiv) was dissolved in DMF (9 mL), followed by HATU (380 mg, 1.0 mmol, 2.5 equiv) and *i*-Pr_2NEt (348 µL, 2.0 mmol, 5.0 equiv). The obtained solution was added to the resin and the suspension was agitated at room temperature for 2 hours. The resin was then washed with DMF (3×) and CH_2Cl_2 (3×) and the loading procedure repeated once. The resin was then dried under vacuum and the loading determined by Fmoc deprotection.

SPPS general protocols

Automated peptide synthesis was carried out on a Biotage Syro Wave™ peptide synthesizer using standard Fmoc/^{t}Bu SPPS chemistry. If not stated differently, SPPS was performed on 0.02 mmol scale using either MeDbz-Gly PHB TentaGel resin or preloaded trityl TentaGel resins (Rapp Polymere). Fmoc deprotection was performed in two stages: piperidine–DMF–formic acid (25:75:0.95, v/v/v) for 3 min, followed by a second treatment for 12 min. The deprotection step was followed by washings with DMF (5×1 min).

Coupling reactions were performed as double couplings using Fmoc-Xaa-OH (6.0 equiv to the resin loading, 0.5 M, dissolved in DMF), HCTU (6.0 equiv, 0.48 M, dissolved in DMF) and *i*-Pr_2NEt (12 equiv, 2.0 M, dissolved in NMP) for 40 min for each coupling (final concentration of Fmoc-Xaa-OH and HCTU = 0.15 M). Couplings reactions for non-standard Fmoc-protected amino acids were performed as outlined for each peptide (see below). General cleavage and deprotection of the peptides was performed by incubating the resin, if not stated differently, with a mixture of TFA–DODT–TIPS (94:3.3:2.7, v/v/v) for 60–90 min. Upon full deprotection (monitored by MALDI-TOF), the reaction mixture was concentrated under a stream of nitrogen and the crude peptide was precipitated by addition of cold diethyl ether. The solid was spun down, washed with cold diethyl ether (2×) and subjected to preparative HPLC purification.

General procedure for thioesterification of peptides from MeDbz-Gly PHB TentaGel resin

After automated peptide elongation, the resin (0.02 mmol, 1.0 equiv) was transferred into a polypropylene syringe equipped with a fritted disk where the resin was washed with CH_2Cl_2 (5×). Activation of the MeDbz linker was performed similarly to a previously reported procedure[3,5]. A solution of 4-nitrophenyl-chloroformate (20 mg, 0.10 mmol, 5.0 equiv) in CH_2Cl_2 (1.0 mL) was added to the resin and the suspension was incubated for 30 min, after which the resin was washed with CH_2Cl_2 (2×). The procedure was repeated once. The resin was then washed with CH_2Cl_2 (5×) and DMF (3×) and a solution of *i*-Pr_2NEt (87 µL, 0.50 mmol, 25.0 equiv) in DMF (1.0 mL) was added to the resin. After 25 min, the resin was washed with DMF (5×). The procedure was repeated once (this procedure was repeated four times for the Int^C-A peptide). The resin was then washed with DMF (5×), *i*-Pr_2NEt in DMF (5%, v/v, 3×) and DMF (5×). To cleave the peptide from the support, the resin was treated with a solution of 3-mercaptopropionic acid ethyl ester (25 µL, 0.20 mmol, 10.0 equiv) and *i*-Pr_2NEt (35 µL, 0.20 mmol, 10.0 equiv) in DMF (1.5 mL). After overnight incubation, the resin was filtered off and washed twice with DMF (1.0 mL). The combined organic phase was concentrated under reduced pressure and then deprotected, if not stated differently, with a mixture of TFA–DODT–TIPS (94:3.3:2.7, v/v/v) for 60–90 min. Upon full deprotection (monitored by MALDI-TOF), the reaction mixture was concentrated under a stream of nitrogen and the crude peptide was precipitated by addition of cold diethyl ether. The solid was spun down, washed with cold diethyl ether (2×) and subjected to preparative HPLC purification.

General procedure for thioesterification of peptides from trityl TentaGel resins

After automated peptide elongation, the resin (0.02 mmol, 1.0 equiv) was transferred into a polypropylene syringe equipped with a fritted disk where the resin was washed with CH_2Cl_2 (5×). The resin was incubated with a solution of HFIP–CH_2Cl_2 (1:4 v/v, 2 mL) for 20 min. The supernatant was collected and the procedure repeated once. The resin was then washed with CH_2Cl_2 (2×) and the combined organic fractions were evaporated under reduced pressure to give the protected peptide as an off-white residue. The thioesterification procedure was performed as previously described[6]. Briefly, the protected peptide was dissolved in anhydrous DMF (1.0 mL) and the obtained solution was cooled to *circa* -30 °C. The thiol of interest (30 equiv) was then added, followed by *i*-Pr_2NEt (5 equiv) and PyBOP (5 equiv). The reaction mixture was stirred at *circa* -30 °C for 3 h, after which it was warmed to room temperature and then concentrated under reduced pressure. The obtained residue was deprotected, if not stated differently, with a mixture of TFA–DODT–TIPS (94:3.3:2.7, v/v/v) for 90 min. Upon full deprotection (monitored by MALDI-TOF), the reaction mixture was concentrated under a stream of nitrogen and the crude peptide was precipitated by addition of cold diethyl ether. The solid was spun down, washed with cold diethyl ether (2×) and subjected to preparative HPLC purification.

General procedure for reduction of oxidized Met containing peptides

Reduction of oxidized methionine residues was performed similarly to a previously described procedure[7]. Briefly, DODT (65 µL, 0.2 M) was added to a solution of the crude peptide in TFA (2.0 mL for a 20 µmol scale), followed by trimethylsilyl bromide (26.4 µL, 0.1 M). The solution was incubated at room temperature for 20 min, after which it was concentrated under a stream of nitrogen. The crude peptide solid was precipitated by addition of cold diethyl ether. The precipitate was spun down, washed with cold diethyl ether and subsequently subjected to preparative HPLC purification.

Alternatively, the reduction could be performed during peptide deprotection under similar conditions: after incubation of full protected peptide with a mixture of TFA–DODT–TIPS (94:3.3:2.7, v/v/v, 4.0 mL) for 90 min, trimethylsilyl bromide (52.8 µL, final concentration 0.1 M) was added and the mixture further incubated for 20 min.

Int^N-B

CISGDSLISLASSGESKDEL

The peptide was synthesized according to the general SPPS protocol outlined above, on Fmoc-Leu PHB TentaGel preloaded resin (0.2 mmol/g). Preparative HPLC purification followed by lyophilization yielded the peptide as a fluffy solid (8.9 mg as a TFA salt; yield 20%).

Prep-HPLC purification conditions (C18 column): 0–10% eluent II in eluent I (5 min gradient) followed by 10–38% eluent II in eluent I (35 min gradient).

Low resolution MS (MALDI-TOF): calc. $[C_{82}H_{140}N_{21}O_{35}S]^+$ $[M + H]^+$: 2010.95 Da; found: 2011.67 Da

HR-MS: calc. $[M + 2H]^{2+}$: 1005.9804 Da; found: 1005.9808 Da

calc. $[M + 3H]^{3+}$: 670.9894 Da; found: 670.9899 Da

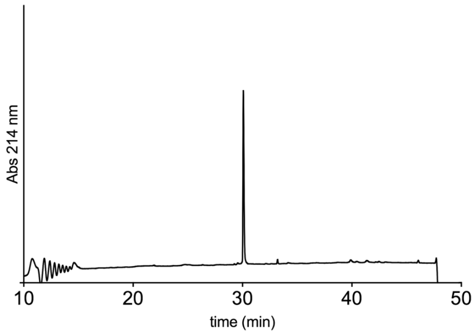

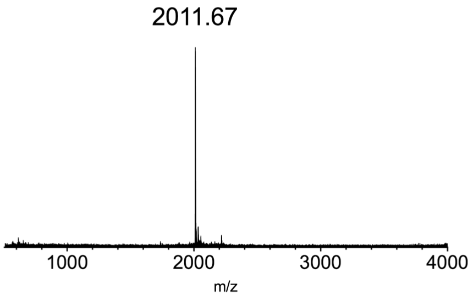

Int^N-B_short

CISGDSLISLA

The peptide was synthesized (2 × 20 µmol scale) according to the general SPPS protocol outlined above, on 2-clorotrityl chloride resin (0.39 mmol/g) that was previously loaded with Fmoc-Ala-OH according to the general procedure outlined above. Deprotection and cleavage of the peptide was performed by incubating the resin with a mixture of TFA–DODT–TIPS (95:2.5:2.5, v/v/v) for 60 min. Preparative HPLC purification followed by lyophilization yielded the peptide as a fluffy solid (13.3 mg as a TFA salt; yield 28%).

Prep-HPLC purification conditions (C18 column): 0–35% eluent II in eluent I (35 min gradient).

Low resolution MS (MALDI-TOF): calc. $[C_{45}H_{80}N_{11}O_{17}S]^{+}$ $[M + H]^{+}$: 1078.54 Da; found: 1078.06 Da

HR-MS: calc. $[M + H]^{+}$: 1078.5449 Da; found: 1078.5412 Da

calc. $[M + 2H]^{2+}$: 539.7761 Da; found: 539.7750 Da

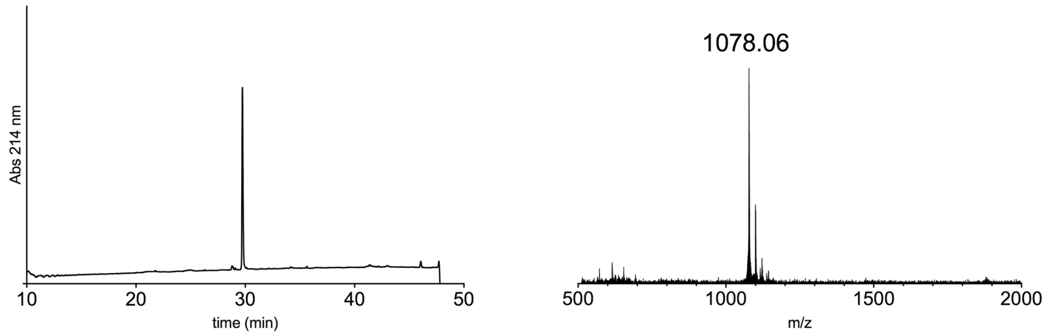

Int^C-A

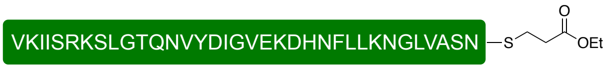

The peptide was synthesized on both preloaded Fmoc-Asn(Trt) trityl TentaGel resin (40 µmol scale, 0.19 mmol/g) and Fmoc-MeDbz-Gly PHB TentaGel resin (20 µmol scale, 0.16 mmol/g) according to the general SPPS protocol outlined above.

When MeDbz-Gly TentaGel resin was used, the loading of the first residue was performed as double coupling using HATU (6.0 equiv) as the coupling reagent and incubating the resin 90 min for each coupling.

Fmoc-DmbGly-OH was incorporated instead of regular Gly at the positions underlined in the sequence (VKIISRKSLGTQNVYDIGVEKDHNFLLKNGLVASN). The coupling was performed as a single coupling similarly to the other coupling steps, but using Fmoc-DmbGly-OH (2.5 equiv), HATU (2.5 equiv) and *i*-Pr_2NEt (5.0 equiv) and incubating the resin

for 90 min. The residue coming after the DmbGly was coupled similarly to the other coupling steps, but using HATU (6.0 equiv) as the coupling reagent.

When MeDbz-Gly TentaGel resin was used, DmbGly was incorporated at the positions underlined in the sequence (VKIISRKSLGTQNVYDIGVEKDHNFLLKNGLVASN).

Boc-Val-OH was coupled to the growing peptide as the N-terminal residue.

After peptide elongation and thioesterification, preparative HPLC purification followed by lyophilization yielded the peptide as a fluffy solid [preloaded Fmoc-Asn(Trt) trityl TentaGel resin (40 µmol): 17.8 mg as a TFA salt; yield 9%. Fmoc-MeDbz-Gly PHB TentaGel resin (20 µmol): 3.4 mg as a TFA salt; yield 3%]

Prep-HPLC purification conditions (C8 column): 0–12% eluent II in eluent I (5 min gradient) followed by 12–33% eluent II in eluent I (35 min gradient).

Low resolution MS (MALDI-TOF): calc. $[C_{177}H_{294}N_{49}O_{53}S]^+$ $[M + H]^+$: 3987.16 Da; found: 3988.34 Da

HR-MS: calc. $[M + 4H]^{4+}$: 997.5446 Da; found: 997.5447 Da

calc. $[M + 5H]^{5+}$: 798.4378 Da; found: 798.4376 Da

calc. $[M + 6H]^{6+}$: 665.5327 Da; found: 665.5326 Da

calc. $[M + 7H]^{7+}$: 570.6005 Da; found: 570.6006 Da

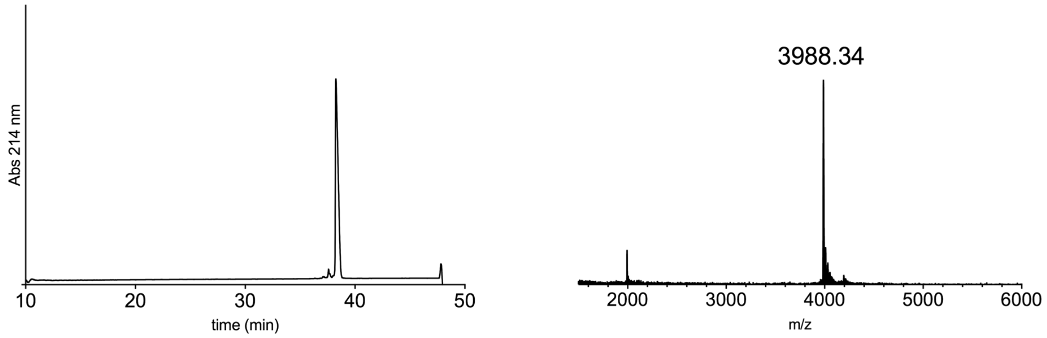

Int^C-A_TFET

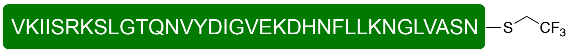

The peptide was synthesized on preloaded Fmoc-Asn(Trt) trityl TentaGel resin (0.19 mmol/g) according to the general SPPS protocol outlined above.

The coupling of the first residue was performed as double coupling using HATU (6.0 equiv) as the coupling reagent for 60 min for each coupling.

Fmoc-DmbGly-OH was incorporated instead of regular Gly at the positions underlined in the sequence (VKIISRKSLGTQNVYDIGVEKDHNFLLKNGLVASN). The coupling was

performed as a single coupling similarly to the other coupling steps, but using Fmoc-DmbGly-OH (2.5 equiv), HATU (2.5 equiv) and *i*-Pr_2NEt (5.0 equiv) and incubating for 90 min. The residue coming after the DmbGly was coupled similarly to the other coupling steps, but using HATU (6.0 equiv) as the coupling reagent.

Boc-Val-OH was coupled to the growing peptide as the N-terminal residue.

After peptide elongation and thioesterification, preparative HPLC purification followed by lyophilization yielded the peptide as a fluffy solid (8.8 mg as a TFA salt; yield 9%).

Prep-HPLC purification conditions (C8 column): 0–10% eluent II in eluent I (5 min gradient) followed by 10–35% eluent II in eluent I (35 min gradient).

Low resolution MS (MALDI-TOF): calc. $[C_{174}H_{287}F_3N_{49}O_{51}S]^+$ $[M + H]^+$: 3969.11 Da; found: 3969.03 Da

HR-MS: calc. $[M + 4H]^{4+}$: 993.0322 Da; found: 993.2817 Da

calc. $[M + 5H]^{5+}$: 794.6273 Da; found: 794.8270 Da

calc. $[M + 6H]^{6+}$: 662.3573 Da; found: 662.5236 Da

calc. $[M + 7H]^{7+}$: 568.0220 Da; found: 568.0215 Da

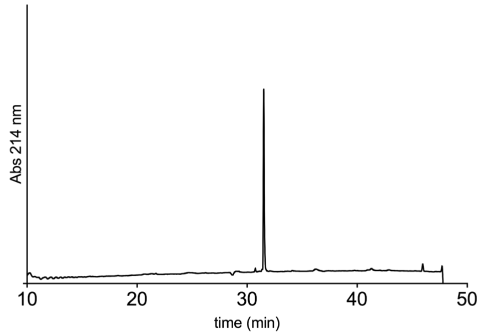

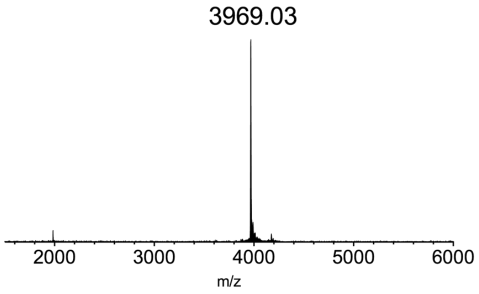

P2X2_WT

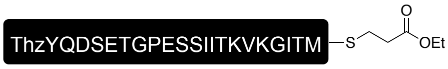

The peptide was synthesized on Fmoc-MeDbz-Gly PHB TentaGel resin (0.16 mmol/g) according to the general SPPS protocol outlined above.

Fmoc-DmbGly-OH was incorporated instead of regular Gly at the position underlined in the sequence (ThzYQDSETGPESSIITKVKGITM). The coupling was performed as a single coupling similarly to the other coupling steps, but using Fmoc-DmbGly-OH (3.0 equiv), HATU (3.0 equiv) and *i*-Pr_2NEt (6.0 equiv) and incubating for 90 min. The residue coming after the DmbGly was coupled similarly to the other coupling steps, but using HATU (6.0 equiv) as the coupling reagent.

Boc-Thz-OH was coupled to the growing peptide as the N-terminal residue.

After peptide elongation, thioesterification and deprotection, the crude peptide was reduced as described above. Preparative HPLC purification followed by lyophilization yielded the peptide as a fluffy solid (4.7 mg as a TFA salt; yield 8%).

Prep-HPLC purification conditions (C8 column): 0–10% eluent II in eluent I (5 min gradient) followed by 10–38% eluent II in eluent I (35 min gradient).

Low resolution MS (MALDI-TOF): calc. $[C_{107}H_{176}N_{25}O_{38}S_3]^+$ $[M + H]^+$: 2516.18 Da; found: 2516.58 Da.

HR-MS: calc. $[M + 2H]^{2+}$: 1258.5936 Da; found: 1258.5945 Da

calc. $[M + 3H]^{3+}$: 839.3981 Da; found: 839.3977 Da

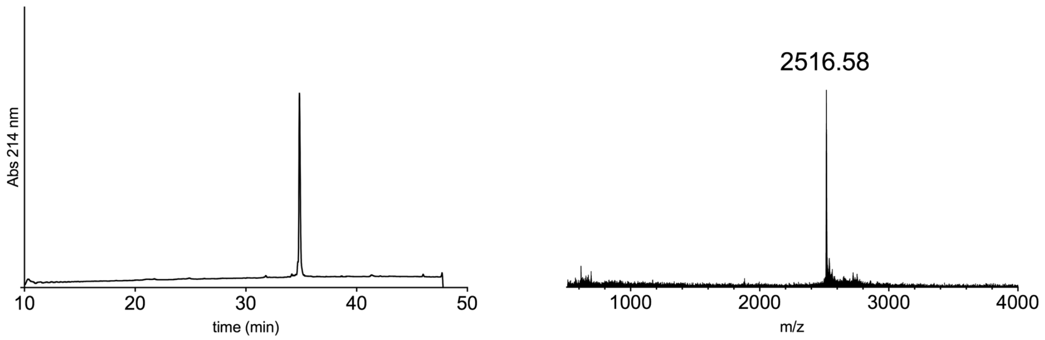

P2X2_hLys71

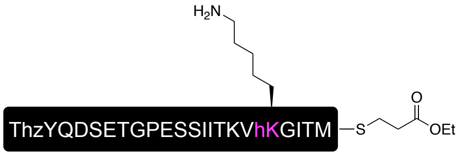

The peptide was synthesized on Fmoc-MeDbz-Gly PHB TentaGel resin (0.16 mmol/g) according to the general SPPS protocol outlined above.

The loading of the first residue was performed as double coupling using HATU (6.0 equiv) as the coupling reagent for 60 min for each coupling. Fmoc-DmbGly-OH was incorporated instead of regular Gly at the position underlined in the sequence (ThzYQDSETGPESSIITKVhKGITM). Homolysine (denoted hK or hLys) was incorporated through a Fmoc/Boc protected amino acid building block. The coupling of Fmoc-DmbGly-OH and Fmoc-hLys(Boc)-OH was performed as a single coupling similarly to the other coupling steps, but using the Fmoc protected amino acid (2.5 equiv), HATU (2.5 equiv) and *i*-Pr_2NEt (5.0 equiv) and incubating for 90 min. The residue coming after the DmbGly was coupled similarly to the other coupling steps, but using HATU (6.0 equiv) as the coupling reagent.

Boc-Thz-OH was coupled to the growing peptide as the N-terminal residue.

After peptide elongation, thioesterification and deprotection, the crude peptide was reduced as described above. Preparative HPLC purification followed by lyophilization yielded the peptide as a fluffy solid (14.4 mg as a TFA salt; yield 12%).

Prep-HPLC purification conditions (C8 column): 0–15% eluent II in eluent I (5 min gradient) followed by 15–35% eluent II in eluent I (32 min gradient).

Low resolution MS (MALDI-TOF): calc. $[C_{108}H_{178}N_{25}O_{38}S_3]^+$ $[M + H]^+$: 2530.19 Da; found: 2529.58 Da.

HR-MS: calc. $[M + 2H]^{2+}$: 1265.6014 Da; found: 1265.6013 Da

calc. $[M + 3H]^{3+}$: 844.0700 Da; found: 844.0699 Da

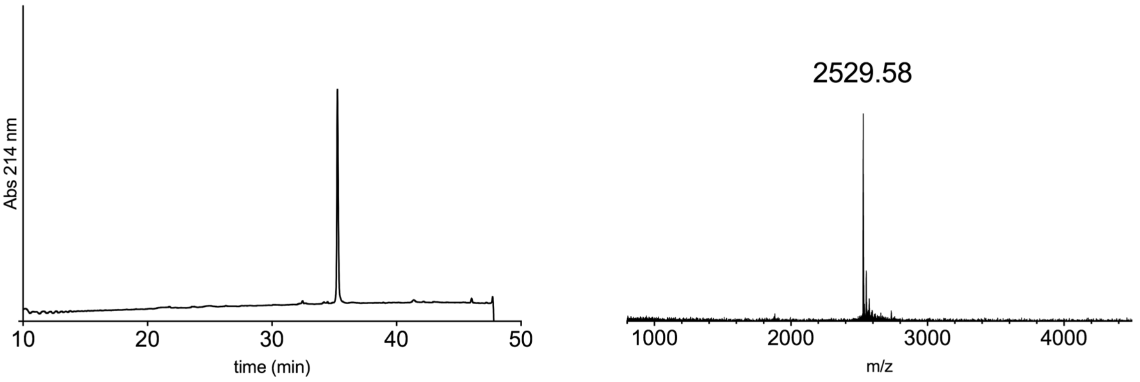

Na_V1.5_DI-DII Linker_NM

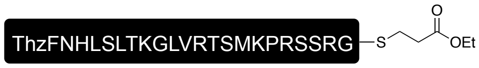

The peptide was synthesized on Fmoc-MeDbz-Gly PHB TentaGel resin (0.16 mmol/g) according to the general SPPS protocol outlined above.

The loading of the first residue was performed as double coupling using HATU (6.0 equiv) as the coupling reagent for 60 min for each coupling.

Boc-Thz-OH was coupled to the growing peptide as the N-terminal residue.

After peptide elongation, thioesterification and deprotection, the crude peptide was reduced as described above. Preparative HPLC purification followed by lyophilization yielded the peptide as a fluffy solid (4.6 mg as a TFA salt; 7% yield).

Prep-HPLC purification conditions (C8 column): 0–10% eluent II in eluent I (5 min gradient) followed by 10–30% eluent II in eluent I (30 min gradient).

Low resolution MS (MALDI-TOF): calc. $[C_{115}H_{196}N_{37}O_{32}S_3]^+$ $[M + H]^+$: 2704.40 Da; found: 2703.35 Da.

HR-MS: calc. $[M + 4H]^{4+}$: 676.8564 Da; found: 676.8557 Da

calc. $[M + 5H]^{5+}$: 541.6866 Da; found: 541.6861 Da

calc. [M + 6H]$^{6+}$: 451.5733 Da; found: 451.5731 Da

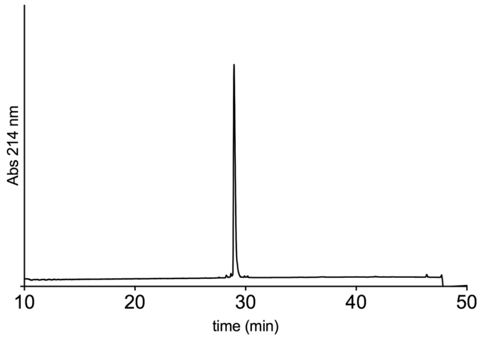

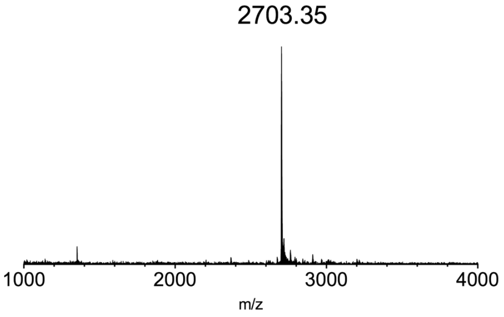

Na$_V$1.5_DI-DII Linker_meArg513

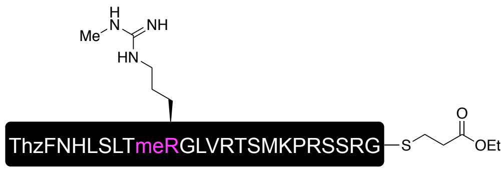

The peptide was synthesized on pre-loaded Fmoc-Gly Trityl TentaGel resin (0.22 mmol/g) according to the general SPPS protocol outlined above.

Methylated arginine (denoted meR or meArg) was incorporated through a Fmoc/Pbf protected amino acid building block. The coupling of Fmoc-Arg(Me,Pbf)-OH was performed as a single coupling similarly to the other coupling steps, but using Fmoc-Arg(Me,Pbf)-OH (2.5 equiv), HATU (2.5 equiv), *i*-Pr_2NEt (5.0 equiv) and incubating for 2 hours.

Boc-Thz-OH was coupled to the growing peptide as the N-terminal residue.

After peptide elongation, thioesterification and deprotection, the crude peptide was reduced as described above. Preparative HPLC purification followed by lyophilization yielded the peptide as a fluffy solid (7.3 mg as a TFA salt; yield 10%).

Prep-HPLC purification conditions (C8 column): 0–10% eluent II in eluent I (5 min gradient) followed by 10–30% eluent II in eluent I (30 min gradient).

Low resolution MS (MALDI-TOF): calc. $[C_{116}H_{198}N_{39}O_{32}S_3]^+$ [M + H]$^+$: 2746.42 Da; found: 2746.66 Da.

HR-MS: calc. [M + 4H]$^{4+}$: 687.3618 Da; found: 687.3608 Da

calc. [M + 5H]$^{5+}$: 550.0909 Da; found: 550.0902 Da

calc. [M + 6H]$^{6+}$: 458.5770 Da; found: 458.5765 Da

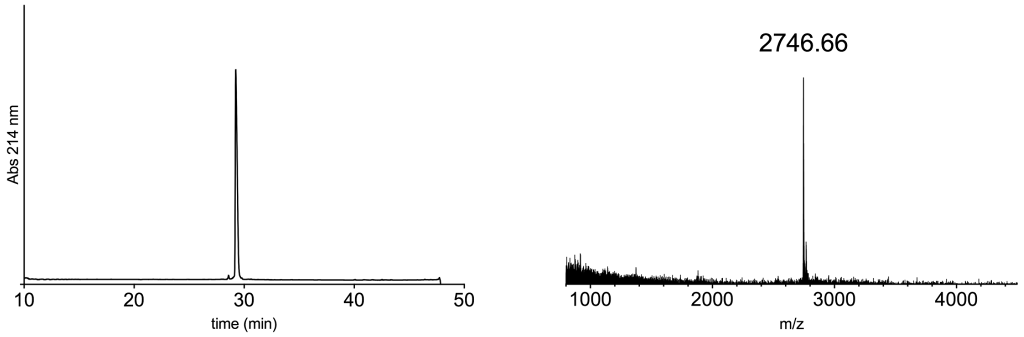

NaV1.5_ DI-DII Linker _phSer516

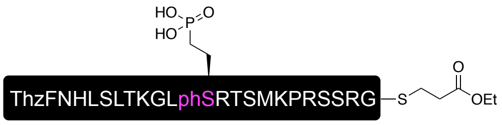

The peptide was synthesized on pre-loaded Fmoc-Gly Trityl TentaGel resin (0.22 mmol/g) according to the general SPPS protocol outlined above.

Phosphonylated serine (denoted phS or phSer) was incorporated through a Fmoc/*ᵗ*Bu protected amino acid building block. The coupling of Fmoc-Pma(*ᵗ*Bu)$_2$-OH was performed as a single coupling similarly to the other coupling steps, but using Fmoc-Pma(*ᵗ*Bu)$_2$-OH (2.5 equiv), HATU (2.5 equiv) and *i*-Pr$_2$NEt (5.0 equiv) and incubating for 2 hours.

Boc-Thz-OH was coupled to the growing peptide as the N-terminal residue.

After peptide elongation, thioesterification and deprotection, the crude peptide was reduced as described above. Preparative HPLC purification followed by lyophilization yielded the peptide as a fluffy solid (7.3 mg as a TFA salt; yield 10%).

Prep-HPLC purification conditions (C8 column): 0–10% eluent II in eluent I (5 min gradient) followed by 10–30% eluent II in eluent I (30 min gradient).

Low resolution MS (MALDI-TOF): calc. $[C_{114}H_{195}N_{37}O_{35}PS_3]^+$ $[M + H]^+$: 2770.35 Da; found: 2771.07 Da.

HR-MS: calc. $[M + 4H]^{4+}$: 693.3441 Da; found: 693.3430 Da

calc. $[M + 5H]^{5+}$: 554.8767 Da; found: 554.8760 Da

calc. $[M + 6H]^{6+}$: 462.5651 Da; found: 462.5646 Da

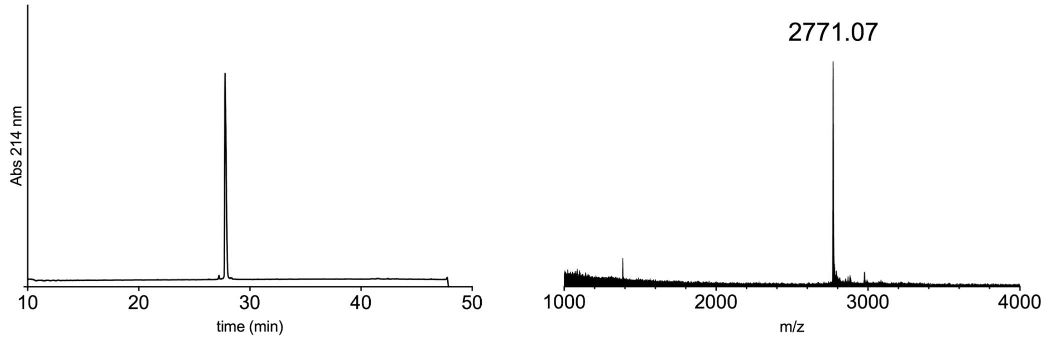

Na$_V$1.5_ DI-DII Linker _meArg513+phSer516

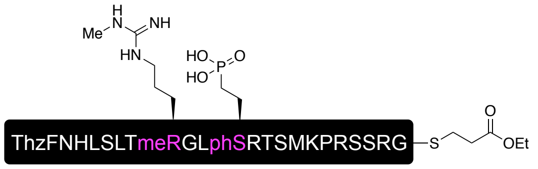

The peptide was synthesized on pre-loaded Fmoc-Gly Trityl TentaGel resin (0.22 mmol/g) according to the general SPPS protocol outlined above.

Phosphonylated serine (denoted phS or phSer) and methylated arginine (denoted meR or meArg) were incorporated through a Fmoc/^{t}Bu and a Fmoc/Pbf protected amino acid building blocks, respectively. The coupling of Fmoc-Arg(Me,Pbf)-OH and Fmoc-Pma(^{t}Bu)$_2$-OH was performed as a single coupling similarly to the other coupling steps, but using the Fmoc protected amino acid (2.5 equiv), HATU (2.5 equiv) and *i*-Pr$_2$NEt (5.0 equiv) and incubating for 2 hours.

Boc-Thz-OH was coupled to the growing peptide as the N-terminal residue.

After peptide elongation, thioesterification and deprotection, the crude peptide was reduced as described above. Preparative HPLC purification followed by lyophilization yielded the peptide as a fluffy solid (9.0 mg as a TFA salt; yield 12%).

Prep-HPLC purification conditions (C8 column): 0–10% eluent II in eluent I (5 min gradient) followed by 10–30% eluent II in eluent I (30 min gradient).

Low resolution MS (MALDI-TOF): calc. $[C_{115}H_{197}N_{39}O_{35}PS_3]^+$ $[M + H]^+$: 2812.38 Da; found: 2812.11 Da.

HR-MS: calc. $[M + 4H]^{4+}$: 703.8495 Da; found: 703.8481 Da

calc. $[M + 5H]^{5+}$: 563.2811 Da; found: 563.2801 Da

calc. $[M + 6H]^{6+}$: 469.5688 Da; found: 469.5681 Da

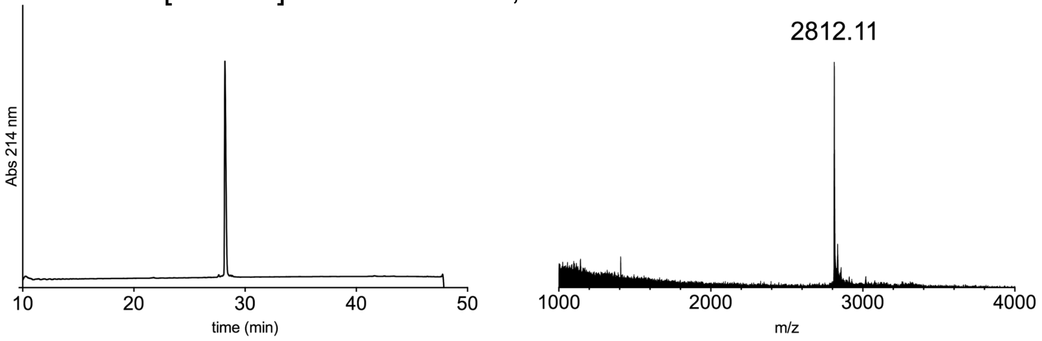

Na$_{V}$1.5_ DIII-DIV Linker _NM

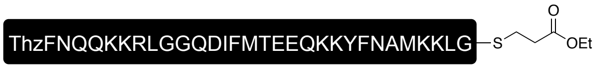

The peptide was synthesized on Fmoc-MeDbz-Gly PHB TentaGel resin (0.16 mmol/g) according to the general SPPS protocol outlined above.

The loading of the first residue was performed as double coupling using HATU (6.0 equiv) as the coupling reagent and incubating for 60 min (first coupling) + 90 min (second coupling).

Fmoc-DmbGly-OH was incorporated instead of regular Gly at the position underlined in the sequence (ThzFNQQKKRLGGQDIFMTEEQKKYFNAMKKLG). The coupling was performed as a single coupling similarly to the other coupling steps, but using Fmoc-DmbGly-OH (3.0 equiv), HATU (3.0 equiv) and *i*-Pr_2NEt (6.0 equiv) and incubating for 90 min. The residue coming after the DmbGly was coupled similarly to the other coupling steps, but using HATU (6.0 equiv) as the coupling reagent.

Boc-Thz-OH was coupled to the growing peptide as the N-terminal residue.

After peptide elongation, thioesterification and deprotection, the crude peptide was subjected to preparative HPLC purification. Lyophilization of the collected fractions yielded the peptide as a fluffy solid (4.0 mg as a TFA salt; yield 4%).

Prep-HPLC purification conditions (C8 column): 0–15% eluent II in eluent I (5 min gradient) followed by 15–36% eluent II in eluent I (30 min gradient).

Low resolution MS (MALDI-TOF): calc. $[C_{170}H_{271}N_{46}O_{47}S_4]^+$ $[M + H]^+$: 3837.91 Da; found: 3839.30 Da.

HR-MS: calc. $[M + 5H]^{5+}$: 768.3886 Da; found: 768.5893 Da

calc. $[M + 6H]^{6+}$: 640.4917 Da; found: 640.6594 Da

calc. $[M + 7H]^{7+}$: 549.1368 Da; found: 549.2803 Da

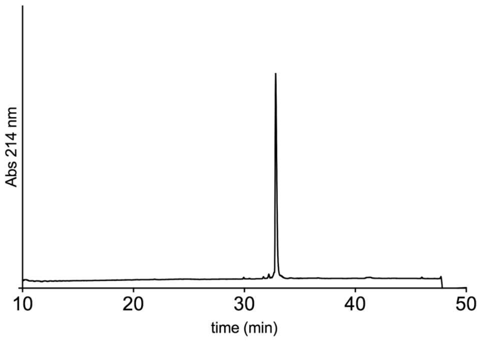

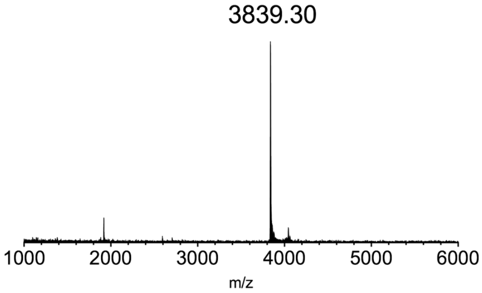

Na$_V$1.5_ DIII-DIV Linker _phTyr1495

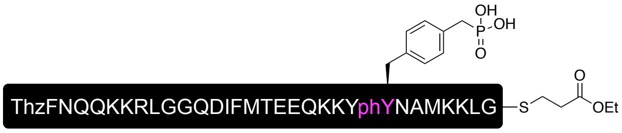

The peptide was synthesized on pre-loaded Fmoc-Gly Trityl TentaGel resin (0.22 mmol/g) according to the general SPPS protocol outlined above.

The loading of the first residue was performed as double coupling using HATU (6.0 equiv) as the coupling reagent and incubating for 60 min.

Phosphonylated tyrosine (denoted phY or phTyr) was incorporated through a Fmoc/^{t}Bu protected amino acid building block. The coupling of Fmoc-Pmp(^{t}Bu)$_2$-OH was performed as a single coupling similarly to the other coupling steps, but using Fmoc-Pmp(^{t}Bu)$_2$-OH (2.5 equiv), HATU (2.5 equiv) and *i*-Pr$_2$NEt (5.0 equiv) and incubating for 2 hours.

Boc-Thz-OH was coupled to the growing peptide as the N-terminal residue.

After peptide elongation, thioesterification and deprotection, the crude peptide was reduced as described above. Preparative HPLC purification followed by lyophilization yielded the peptide as a fluffy solid (9.0 mg as a TFA salt; yield 9%).

Prep-HPLC purification conditions (C8 column): 0–15% eluent II in eluent I (5 min gradient) followed by 15–36% eluent II in eluent I (30 min gradient).

Low resolution MS (MALDI-TOF): calc. $[C_{171}H_{274}N_{46}O_{50}PS_4]^+$ $[M + H]^+$: 3931.90 Da; found: 3932.64 Da.

HR-MS: calc. $[M + 5H]^{5+}$: 787.1850 Da; found: 787.3862 Da

calc. $[M + 6H]^{6+}$: 656.1554 Da; found: 656.3225 Da

calc. $[M + 7H]^{7+}$: 562.7061 Da; found: 562.7064 Da

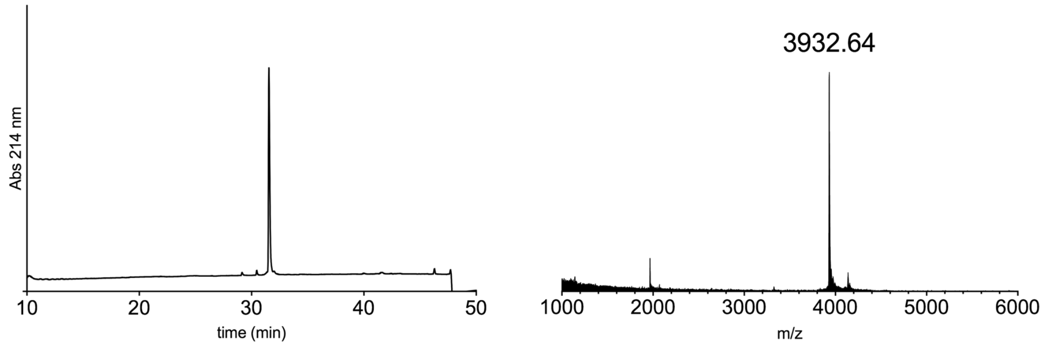

Na_V1.5_ DIII-DIV Linker _tAcLys1479

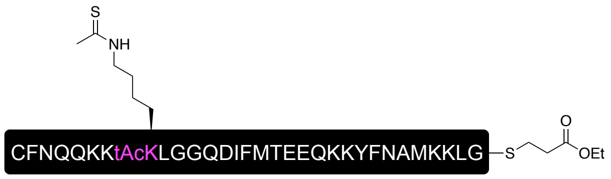

The peptide was synthesized on pre-loaded Fmoc-Gly Trityl TentaGel resin (0.21 mmol/g) according to the general SPPS protocol outlined above.

The loading of the first residue was performed as double coupling using HATU (6.0 equiv) as the coupling reagent and incubating for 60 min.

Thioacetylated lysine (denoted tAcK or tAcLys) was incorporated through a Fmoc protected amino acid building block. The coupling of Fmoc-tAcLys-OH was performed as a single coupling similarly to the other coupling steps, but by using Fmoc-tAcLys-OH (2.5 equiv), HATU (2.5 equiv) and *i*-Pr_2NEt (5.0 equiv) and incubating for 2 hours.

Boc-Cys(Trt)-OH was coupled to the growing peptide as the N-terminal residue.

After peptide elongation, thioesterification and deprotection, the crude peptide was reduced as described above. Preparative HPLC purification followed by lyophilization yielded the peptide as a fluffy solid (7.0 mg as a TFA salt; yield 7%).

Prep-HPLC purification conditions (C8 column): 0–15% eluent II in eluent I (5 min gradient) followed by 15–35% eluent II in eluent I (30 min gradient).

Low resolution MS (MALDI-TOF): calc. $[C_{171}H_{273}N_{44}O_{47}S_5]^+$ $[M + H]^+$: 3855.90 Da; found: 3856.23 Da.

HR-MS: calc. $[M + 4H]^{4+}$: 964.7294 Da; found: 964.9812 Da

calc. $[M + 5H]^{5+}$: 771.9849 Da; found: 772.1857 Da

calc. $[M + 6H]^{6+}$: 643.4887 Da; found: 643.6558 Da

calc. $[M + 7H]^{7+}$: 551.8489 Da; found: 551.8490 Da

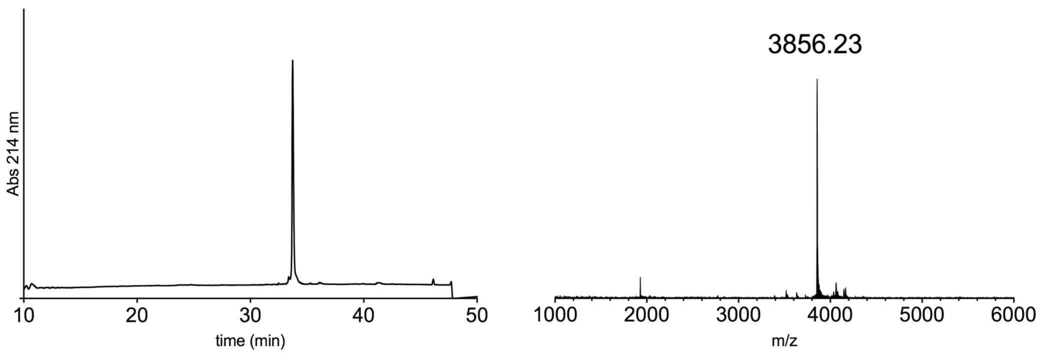

Na$_V$1.5_ DIII-DIV Linker _ tAcLys1479 + phTyr1495

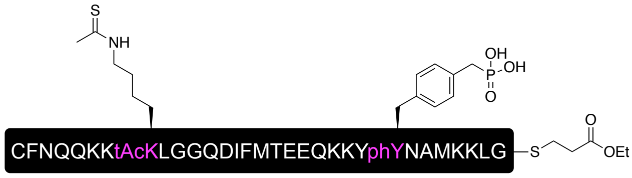

The peptide was synthesized on pre-loaded Fmoc-Gly Trityl TentaGel resin (0.21 mmol/g) according to the general SPPS protocol outlined above.

Phosphonylated tyrosine (denoted phY or phTyr) and thioacetylated lysine (denoted tAcK or tAcLys) were incorporated through a Fmoc/^{t}Bu and a Fmoc protected amino acid building blocks, respectively. The coupling of Fmoc-Pmp(^{t}Bu)$_2$-OH and Fmoc-tAcLys-OH was performed as a single coupling similarly to the other coupling steps, but by using the Fmoc protected amino acid (2.5 equiv), HATU (2.5 equiv) and *i*-Pr$_2$NEt (5.0 equiv) and incubating for 2 hours.

Boc-Cys(Trt)-OH was coupled to the growing peptide as the N-terminal residue.

After peptide elongation, thioesterification and deprotection, the crude peptide was reduced as described above. Preparative HPLC purification followed by lyophilization yielded the peptide as a fluffy solid (4.3 mg as a TFA salt; yield 4%).

Prep-HPLC purification conditions (C8 column): 0–15% eluent II in eluent I (5 min gradient) followed by 15–35% eluent II in eluent I (30 min gradient).

Low resolution MS (MALDI-TOF): calc. $[C_{172}H_{276}N_{44}O_{50}PS_5]^+$ $[M + H]^+$: 3949.88 Da; found: 3951.86 Da.

HR-MS: calc. $[M + 4H]^{4+}$: 988.2249 Da; found: 988.4745 Da

calc. $[M + 5H]^{5+}$: 790.7813 Da; found: 790.9813 Da

calc. $[M + 6H]^{6+}$: 659.3195 Da; found: 659.3189 Da

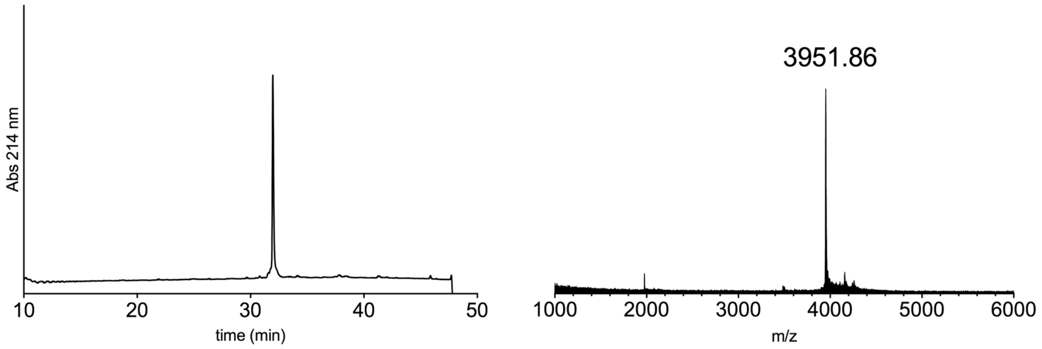

General procedure for one-pot ligation of peptide fragments_C to N directed ligations

Before performing the ligation, the buffer (guanidinium chloride 6M, Na_2HPO_4 100mM) was sparged with nitrogen. TCEP·HCl was then dissolved in buffer (20 mM), followed by addition of appropriate thiol (40 mM MPAA or 2% v/v TFET[8], see below for details). The solution was adjusted to pH ~7 with NaOH 5M and the ion channel peptide fragment (0.57 µmol, 1.0 equiv, 2 mM) was then added, followed by either the Int^N-B or Int^N-B_short peptide fragment (0.57 µmol, 1.0 equiv, see details below). The pH was readjusted to ~7 and incubated at 37 °C. After conversion to the desired ligated product (monitored by HPLC and MALDI-TOF), TCEP·HCl (to reach 40 mM final concentration) and $MeONH_2$·HCl (to reach 200 mM final concentration) were dissolved in buffer (15 µL) and added to the ligation mixture, which was then incubated at 37 °C. After conversion to the desired unmasked N-terminal cysteine peptide (monitored by HPLC and MALDI-TOF), the pH was readjusted to ~7. Activating thiol (40 mM MPAA or 1% v/v TFET, see details below) and the Int^C-A peptide fragment (0.57 µmol, 1.0 equiv if not stated differently, see below for details) were then added. The pH was adjusted to ~7 and the reaction was incubated at either 37 °C or at room temperature (see details below). After conversion to the desired ligated product, the ligation mixture was subjected to preparative HPLC purification.

General procedure for one-pot ligation of peptide fragments_N to C directed ligations

Before performing the ligation, the buffer (guanidinium chloride 6M, Na_2HPO_4 100mM) was sparged with nitrogen. TCEP·HCl was then dissolved in buffer (20 mM) and the pH adjusted to ~6.4 with NaOH 1M. The ion channel peptide fragment (0.57 µmol, 1.0 equiv, 2 mM) was then added, followed by Int^C-A_TFET peptide fragment (0.57 µmol, 1.0 equiv). The pH was readjusted to pH ~6.4 and the reaction mixture was incubated at room temperature. After conversion to the desired ligated product (monitored by HPLC and MALDI-TOF), Int^N-B_short peptide fragment (0.57 µmol, 1.0 equiv) was added, followed by TFET (1% v/v). The pH was adjusted to ~7 and the reaction was incubated at room temperature. After conversion to the desired ligated product, the ligation mixture was subjected to preparative HPLC purification.

Peptide X_{P2X2_WT}

VKIISRKSLGTQNVYDIGVEKDHNFLLKNGLVASN–CYQDSETGPESSIITKVKGITM–CISGDSLISLASSGESKDEL

The full peptide was assembled according to the general procedure "C to N directed ligations" described above, using MPAA as the activating thiol. For the second ligation the N-terminal thioester fragment was used in slight excess (1.1 equiv) and the reaction mixture was incubated at 37 °C. Preparative HPLC purification followed by lyophilization yielded the peptide as a fluffy solid (1.7 mg as a TFA salt; yield 32%).

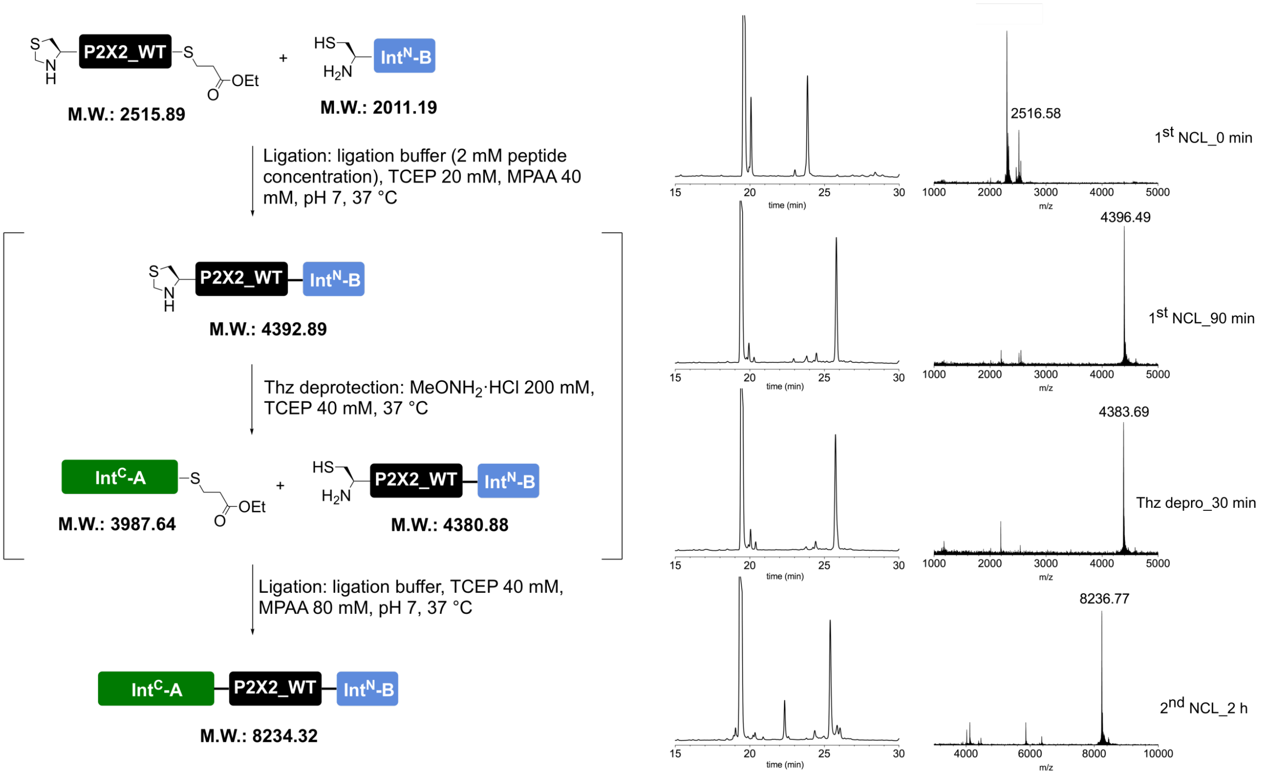

Prep-HPLC purification conditions (C8 column): 0–15% eluent II in eluent I (5 min gradient) followed by 15–45% eluent II in eluent I (45 min gradient).

Low resolution MS (MALDI-TOF): calc. $[C_{355}H_{588}N_{95}O_{122}S_3]^+$ $[M + H]^+$: 8233.20 Da; found: 8239.33 Da.

HR-MS: calc. $[M + 7H]^{7+}$: 1177.1778 Da; found: 1177.3197 Da

calc. $[M + 8H]^{8+}$: 1030.1565 Da; found: 1030.1556 Da

calc. $[M + 9H]^{9+}$: 915.8066 Da; found: 915.9171 Da

calc. $[M + 10H]^{10+}$: 824.3266 Da; found: 824.4266 Da

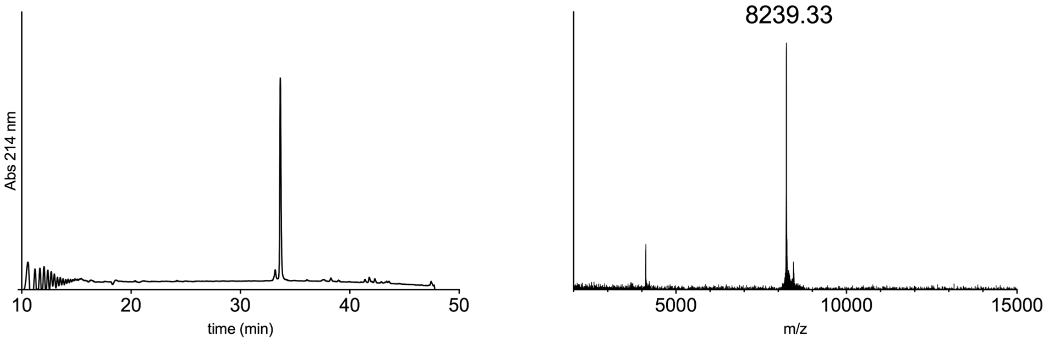

Peptide X_{P2X2_hLys71}

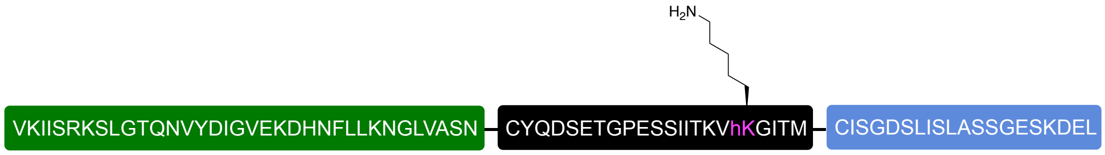

The full peptide was assembled according to the general procedure “C to N directed ligations” described above, using MPAA as the activating thiol. For the second ligation the Int^{C}-A fragment was used in slight excess (1.1 equiv) and the reaction mixture was incubated at 37 °C. Preparative HPLC purification followed by lyophilization yielded the peptide as a fluffy solid (1.9 mg as a TFA salt; yield 35%).

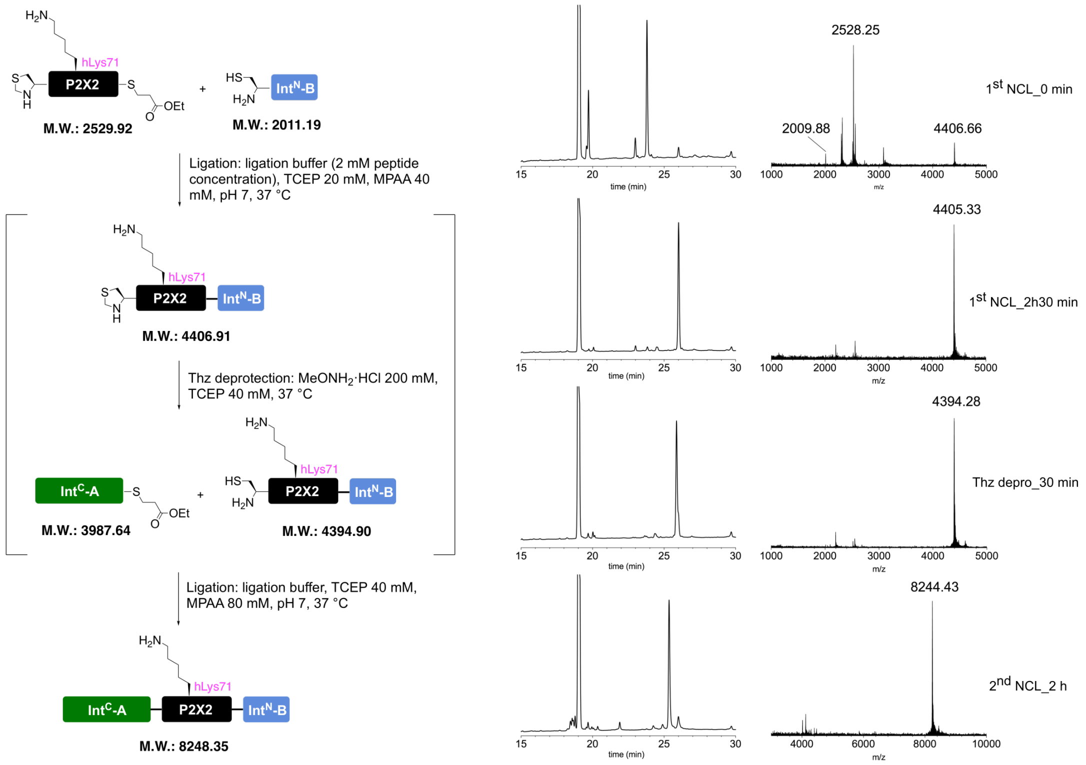

Prep-HPLC purification conditions (C8 column): 0–15% eluent II in eluent I (5 min gradient) followed by 15–45% eluent II in eluent I (45 min gradient).

Low resolution MS (MALDI-TOF): calc. $[C_{356}H_{590}N_{95}O_{122}S_3]^+$ $[M + H]^+$: 8247.21 Da; found: 8251.23 Da.

HR-MS: calc. $[M + 7H]^{7+}$: 1179.1801 Da; found: 1179.1802 Da

calc. $[M + 8H]^{8+}$: 1031.9084 Da; found: 1032.0340 Da

calc. $[M + 9H]^{9+}$: 917.3638 Da; found: 917.3645 Da

calc. $[M + 10H]^{10+}$: 825.7282 Da; found: 825.7285 Da

calc. $[M + 11H]^{11+}$: 750.7535 Da; found: 750.7541 Da

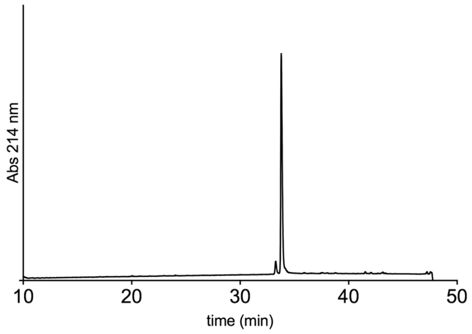

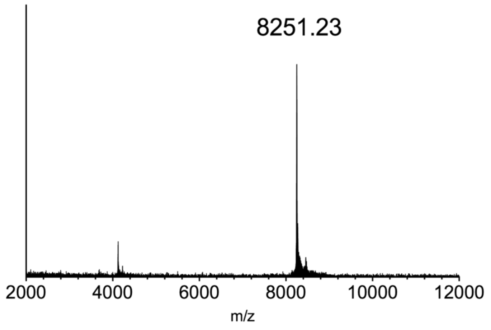

Peptide $X_{Nav1.5\_DI\text{-}DII\ Linker\_NM}$

VKIISRKSLGTQNVYDIGVEKDHNFLLKNGLVASN–CFNHLSLTKGLVRTSMKPRSSRG–CISGDSLISLA

The full peptide was assembled according to the general procedure "C to N directed ligations" described above, using TFET as the activating thiol. For the second ligation, the reaction mixture was incubated at room temperature. Preparative HPLC purification followed by lyophilization yielded the peptide as a fluffy solid (1.8 mg as a TFA salt; yield 35%).

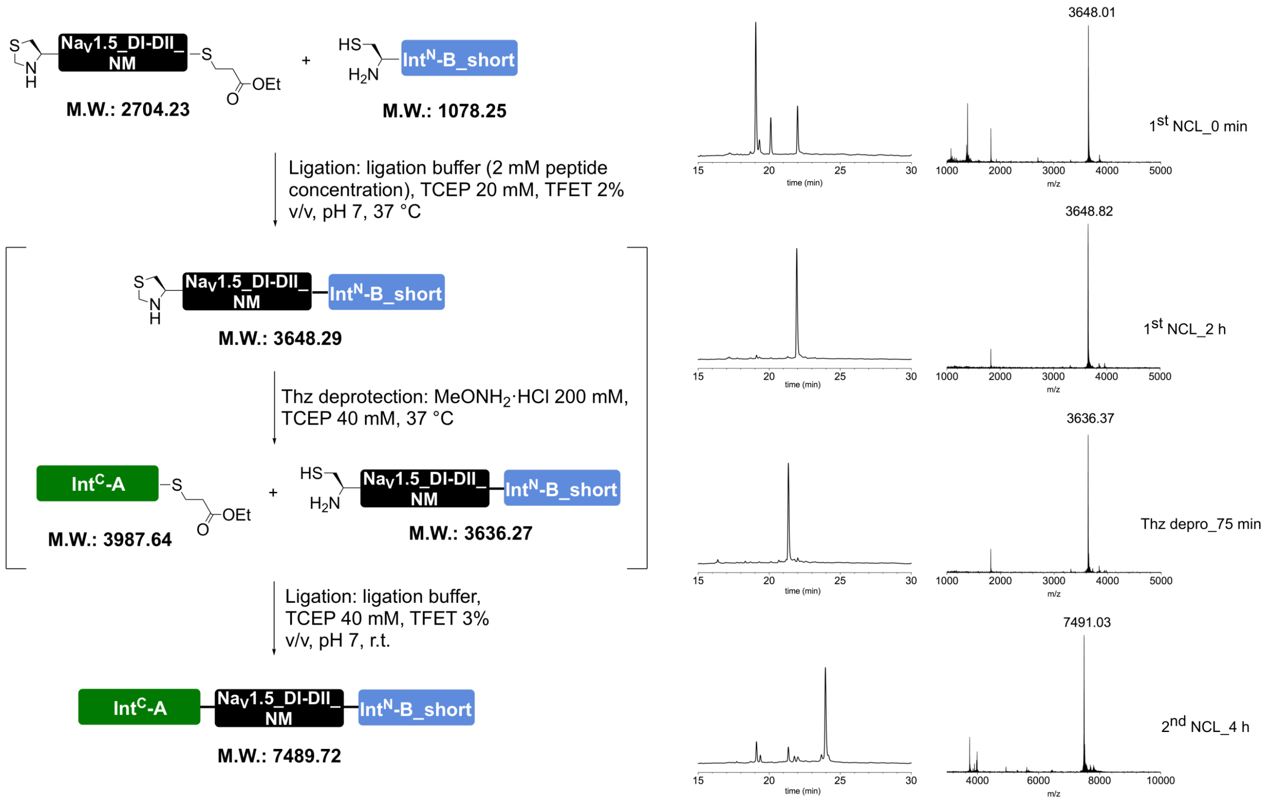

Prep-HPLC purification conditions (C8 column): 0–10% eluent II in eluent I (5 min gradient) followed by 10–45% eluent II in eluent I (45 min gradient).

Low resolution MS (MALDI-TOF): calc. $[C_{326}H_{548}N_{97}O_{98}S_3]^+$ $[M + H]^+$: 7489.01 Da; found: 7487.79 Da.

HR-MS: calc. $[M + 7H]^{7+}$: 1070.7224 Da; found: 1070.8655 Da

calc. $[M + 8H]^{8+}$: 937.0080 Da; found: 937.1330 Da

calc. $[M + 9H]^{9+}$: 833.1194 Da; found: 833.1187 Da

calc. $[M + 10H]^{10+}$: 749.9081 Da; found: 749.9077 Da

calc. $[M + 11H]^{11+}$: 681.8262 Da; found: 681.8260 Da

calc. $[M + 12H]^{12+}$: 625.0913 Da; found: 625.0910 Da

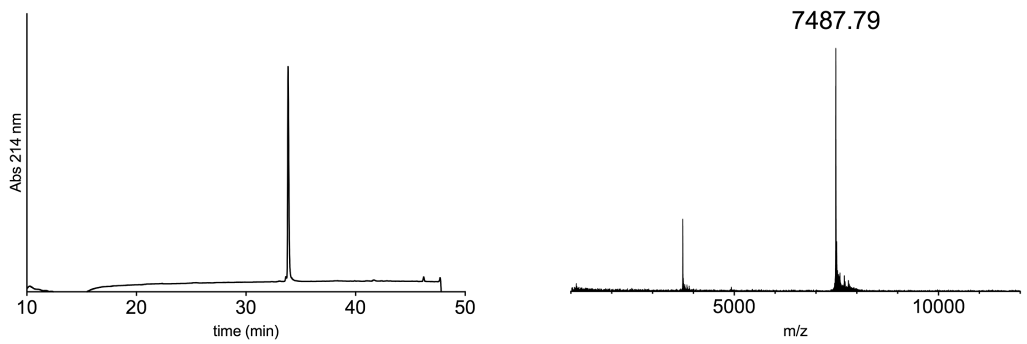

Peptide $X_{Nav1.5\_DI\text{-}DII\ Linker\_meArg513}$

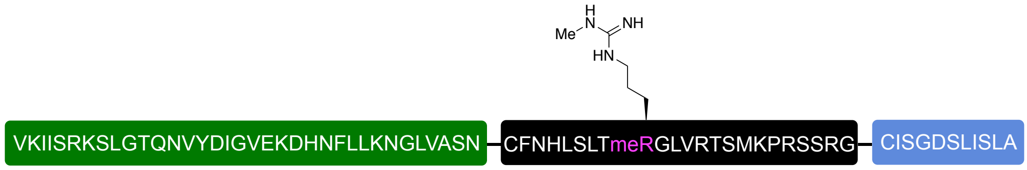

The full peptide was assembled according to the general procedure "C to N directed ligations" described above, using MPAA as the activating thiol. For the second ligation, the reaction mixture was incubated at room temperature. Since the desired product co-eluted with MPAA during preparative HPLC purification, the isolated fraction containing the peptide was dialyzed in multiple steps (2h + 2h + over night at 5 ºC) in water using a cellulose membrane with a cutoff of 2 kDa. The dialyzed fraction was then diluted with eluent I and lyophilized to obtain the peptide as a fluffy solid (1.0 mg as a TFA salt; yield 19%).

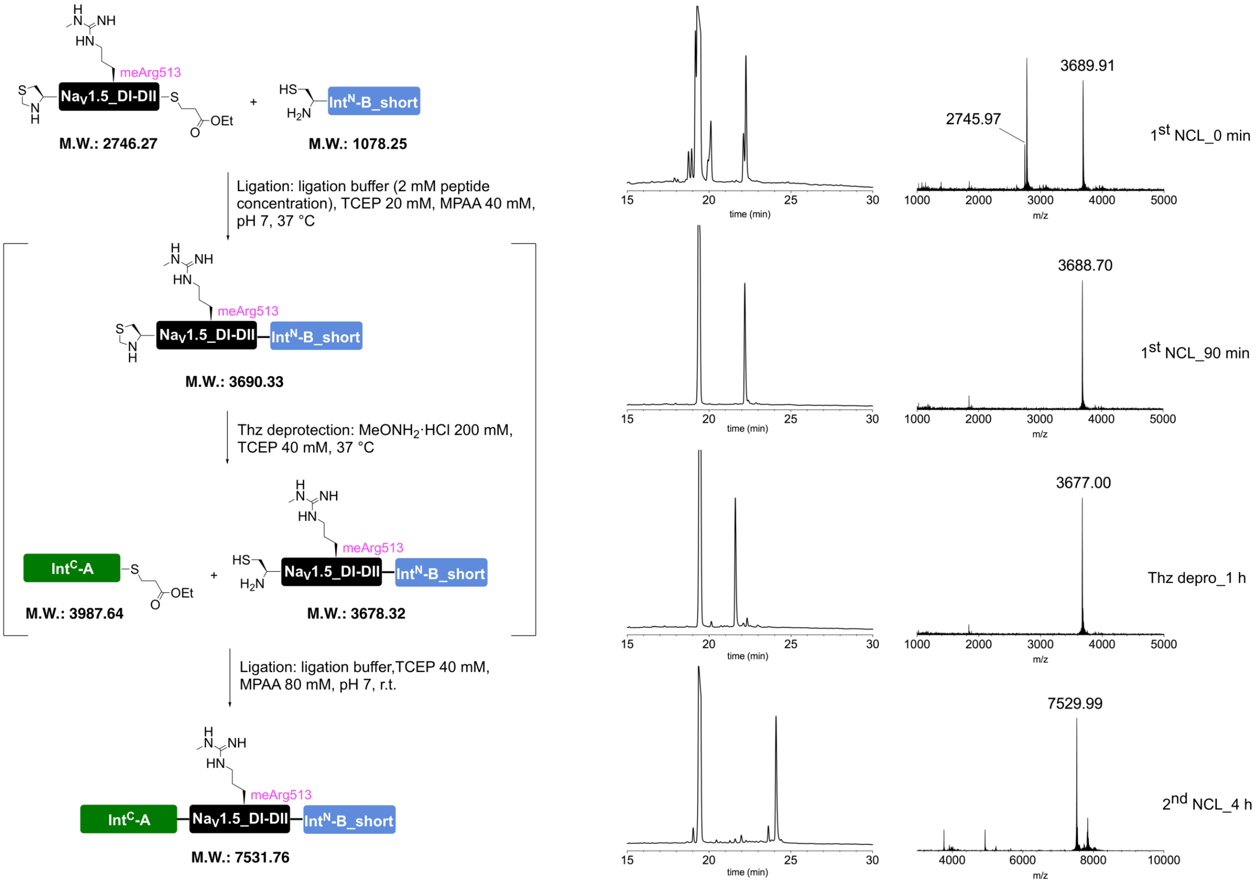

Prep-HPLC purification conditions (C8 column): 0–10% eluent II in eluent I (5 min gradient) followed by 10–45% eluent II in eluent I (45 min gradient).

Low resolution MS (MALDI-TOF): calc. $[C_{327}H_{550}N_{99}O_{98}S_3]^+$ $[M + H]^+$: 7531.03 Da; found: 7529.99 Da.

HR-MS: calc. $[M + 7H]^{7+}$: 1076.7255 Da; found: 1076.8680 Da

calc. $[M + 8H]^{8+}$: 942.3861 Da; found: 942.3846 Da

calc. $[M + 9H]^{9+}$: 837.7885 Da; found: 837.7875 Da

calc. $[M + 10H]^{10+}$: 754.1103 Da; found: 754.1096 Da

calc. $[M + 11H]^{11+}$: 685.6464 Da; found: 685.6457 Da

calc. $[M + 12H]^{12+}$: 628.5931 Da; found: 628.5927 Da

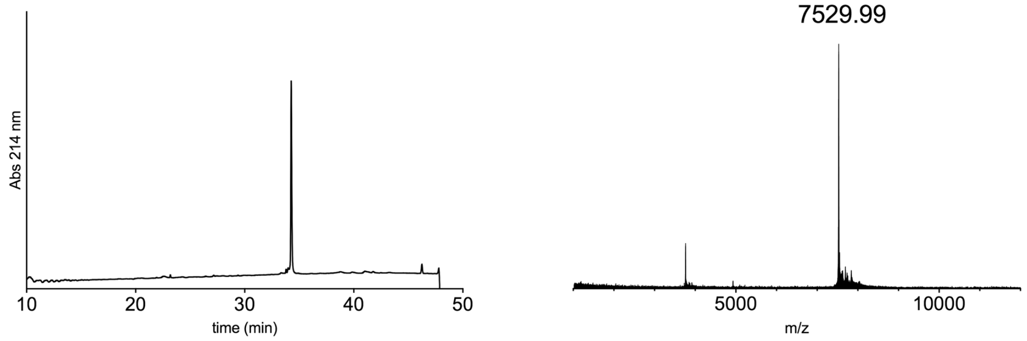

Peptide X$_{\text{Nav1.5\_DI-DII Linker\_phSer516}}$

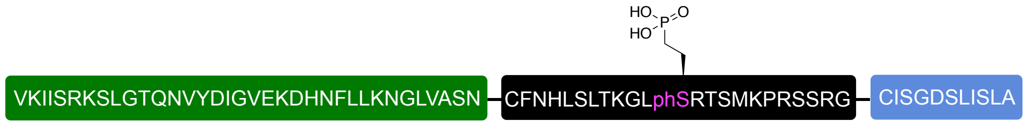

The full peptide was assembled according to the general procedure “C to N directed ligations” described above, using TFET as the activating thiol. For the second ligation, the reaction mixture was incubated at room temperature. Preparative HPLC purification followed by lyophilization yielded the peptide as a fluffy solid (1.7 mg as a TFA salt; yield 33%).

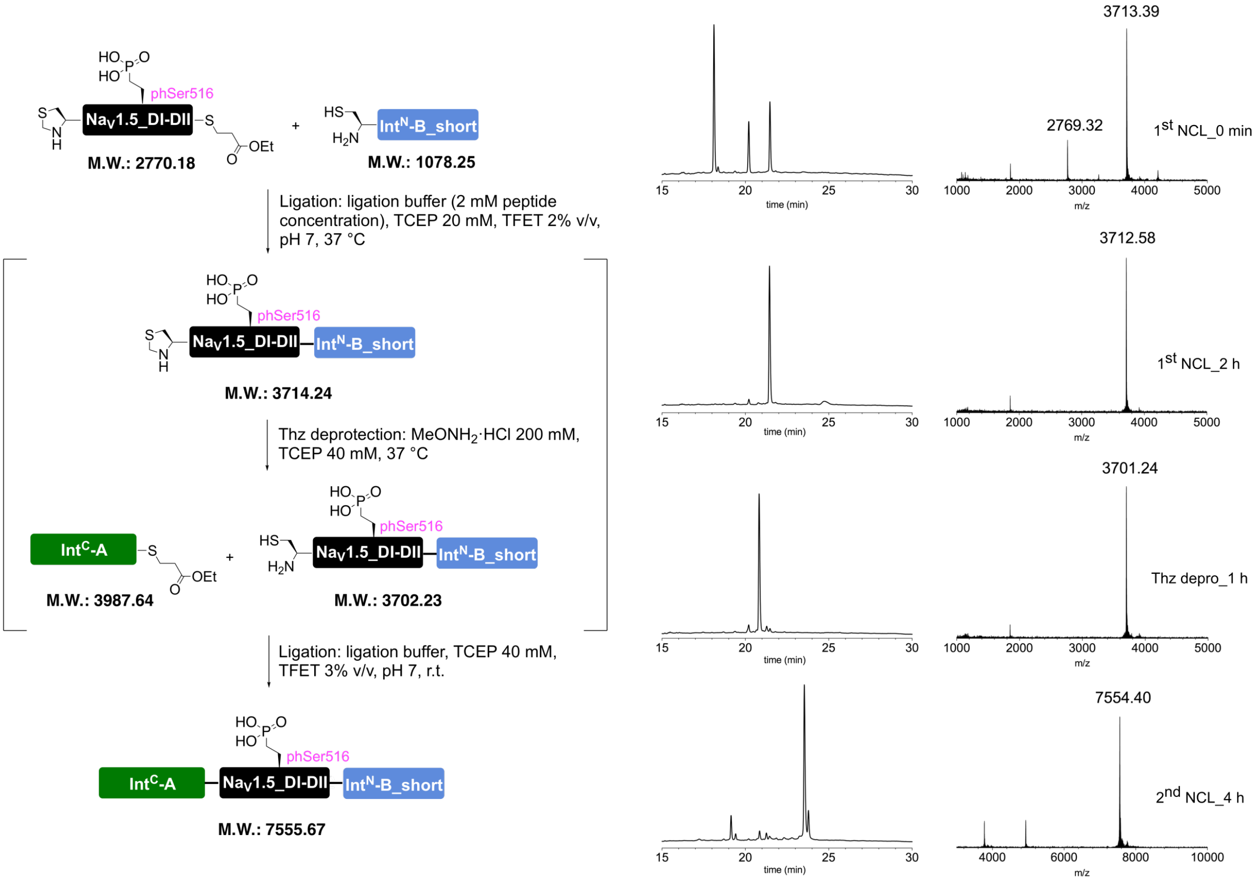

Prep-HPLC purification conditions (C8 column): 0–10% eluent II in eluent I (5 min gradient) followed by 10–45% eluent II in eluent I (45 min gradient).

Low resolution MS (MALDI-TOF): calc. $[C_{325}H_{547}N_{97}O_{101}PS_3]^+$ $[M + H]^+$: 7554.96 Da; found: 7553.58 Da.

HR-MS: calc. $[M + 7H]^{7+}$: 1080.1440 Da; found: 1080.2856 Da

calc. $[M + 8H]^{8+}$: 945.2519 Da; found: 945.3761 Da

calc. $[M + 9H]^{9+}$: 840.4472 Da; found: 840.4463 Da

calc. $[M + 10H]^{10+}$: 756.5032 Da; found: 756.5023 Da

calc. $[M + 11H]^{11+}$: 687.8217 Da; found: 687.8210 Da

calc. $[M + 12H]^{12+}$: 630.5872 Da; found: 630.5868 Da

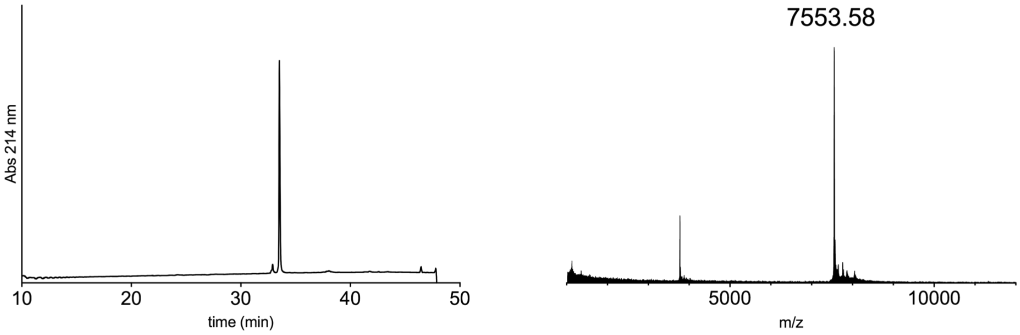

Peptide $X_{Nav1.5\_DI\text{-}DII\ Linker\_meArg513\ +\ phSer516}$

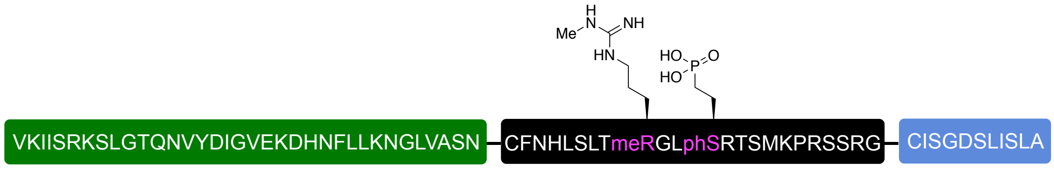

The full peptide was assembled according to the general procedure "C to N directed ligations" described above, using TFET as the activating thiol. For the second ligation, the reaction mixture was incubated at room temperature. Preparative HPLC purification followed by lyophilization yielded the peptide as a fluffy solid (1.7 mg as a TFA salt; yield 33%).

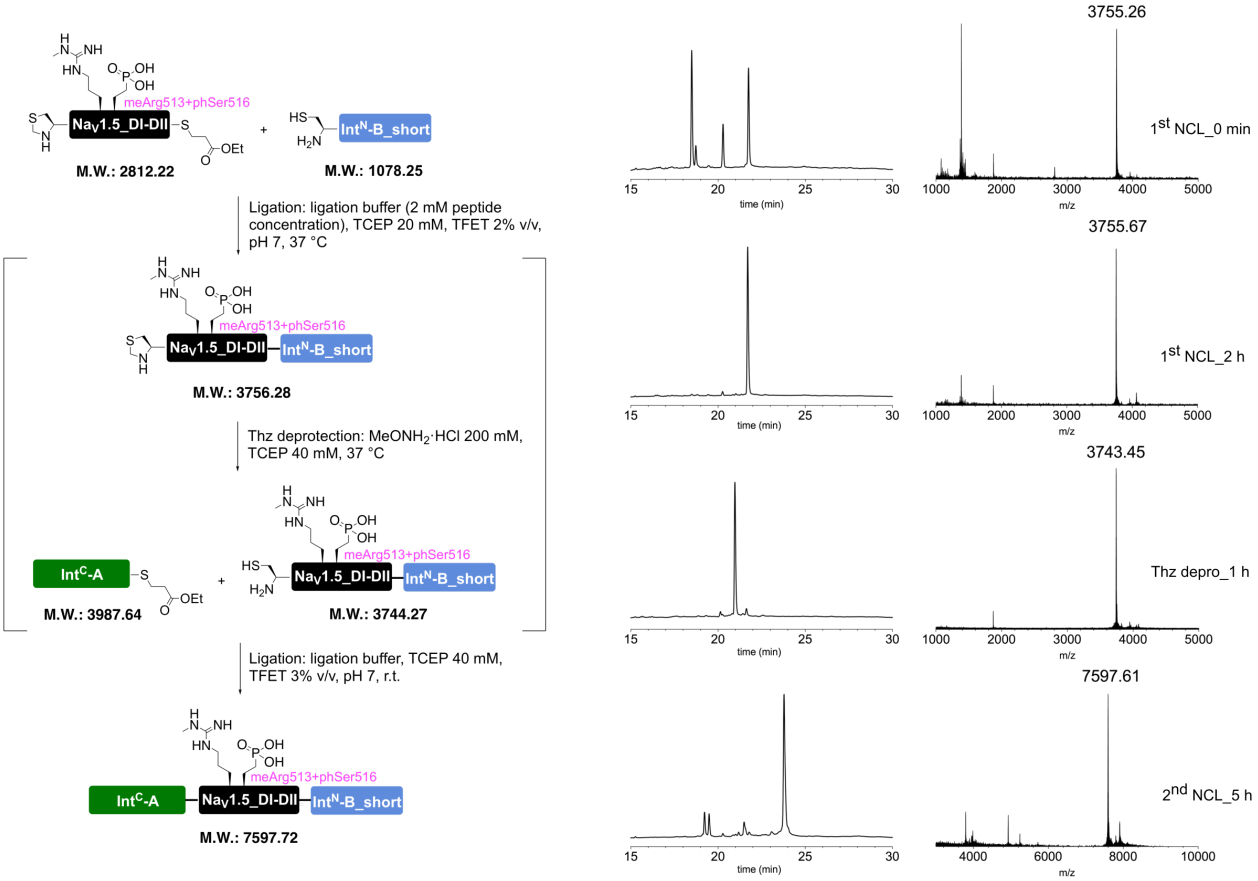

Prep-HPLC purification conditions (C8 column): 0–10% eluent II in eluent I (5 min gradient) followed by 10–45% eluent II in eluent I (45 min gradient).

Low resolution MS (MALDI-TOF): calc. $[C_{326}H_{549}N_{99}O_{101}PS_3]^+$ $[M + H]^+$: 7596.99 Da; found: 7595.97 Da.

HR-MS: calc. $[M + 7H]^{7+}$: 1086.1471 Da; found: 1086.2893 Da

calc. $[M + 8H]^{8+}$: 950.6300 Da; found: 950.6293 Da

calc. $[M + 9H]^{9+}$: 845.1163 Da; found: 845.1160 Da

calc. $[M + 10H]^{10+}$: 760.7054 Da; found: 760.7048 Da

calc. $[M + 11H]^{11+}$: 691.6419 Da; found: 691.6417 Da

calc. $[M + 12H]^{12+}$: 634.0890 Da; found: 634.0889 Da

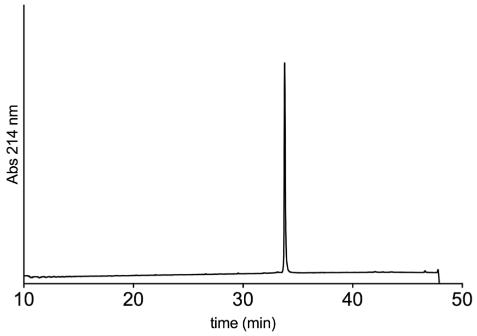

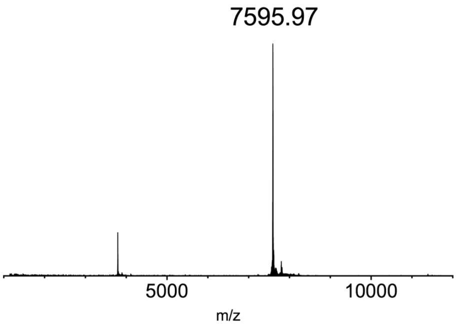

Peptide $X_{Nav1.5\_DIII\text{-}DIV\ Linker\_NM}$

VKIISRKSLGTQNVYDIGVEKDHNFLLKNGLVASN–CFNQQKKRLGGQDIFMTEEQKKYFNAMKKLG–CISGDSLISLA

The full peptide was assembled according to the general procedure “C to N directed ligations” described above, using TFET as the activating thiol. For the second ligation, the reaction mixture was incubated at room temperature. Preparative HPLC purification followed by lyophilization yielded the peptide as a fluffy solid (0.9 mg as a TFA salt; yield 15%).

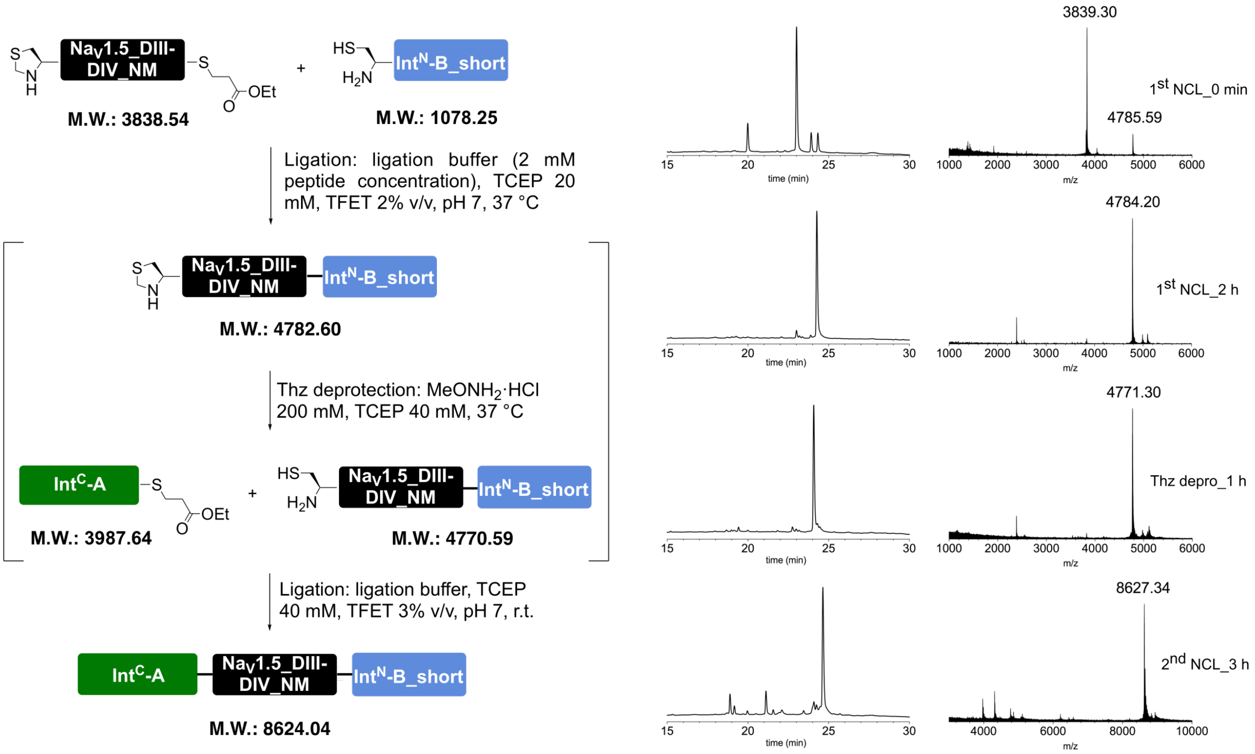

Prep-HPLC purification conditions (C8 column): 0–10% eluent II in eluent I (5 min gradient) followed by 10–45% eluent II in eluent I (45 min gradient).

Low resolution MS (MALDI-TOF): calc. $[C_{381}H_{623}N_{106}O_{113}S_4]^+$ $[M + H]^+$: 8623.53 Da; found: 8622.99 Da.

HR-MS: calc. $[M + 8H]^{8+}$: 1078.8222 Da; found: 1078.8195 Da

calc. $[M + 9H]^{9+}$: 959.0650 Da; found: 959.1741 Da

calc. $[M + 10H]^{10+}$: 863.2592 Da; found: 863.3580 Da

calc. $[M + 11H]^{11+}$: 784.8726 Da; found: 784.8713 Da

calc. $[M + 12H]^{12+}$: 719.5505 Da; found: 719.5495 Da

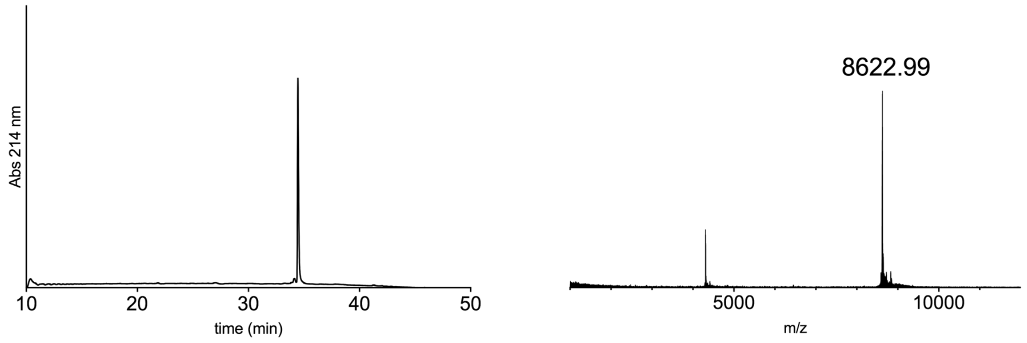

Peptide X$_{Nav1.5_DIII\text{-}DIV\ Linker_phTyr1495}$

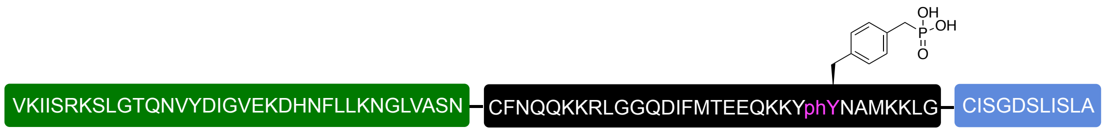

The full peptide was assembled according to the general procedure "C to N directed ligations" described above, using TFET as the activating thiol. For the second ligation, the reaction mixture was incubated at room temperature. Preparative HPLC purification followed by lyophilization yielded the peptide as a fluffy solid (2.0 mg as a TFA salt; yield 34%).

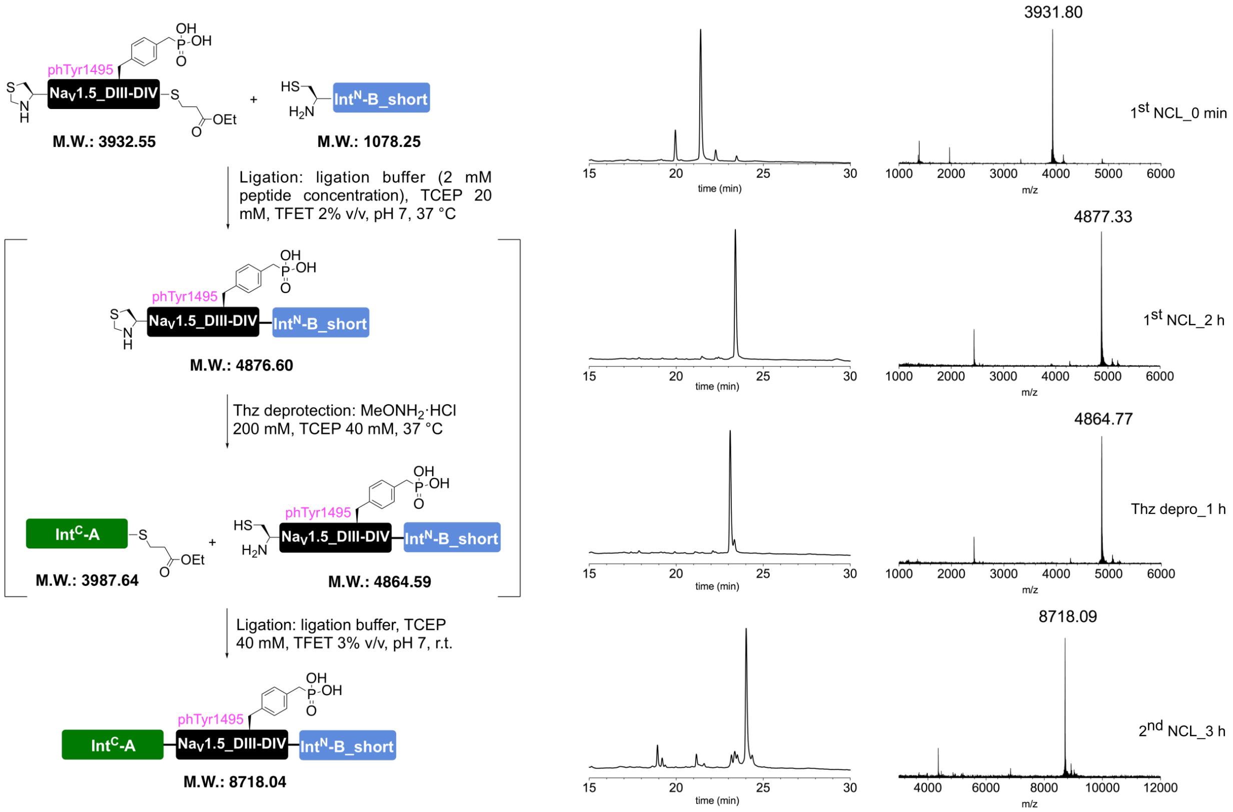

Prep-HPLC purification conditions (C8 column): 0–10% eluent II in eluent I (5 min gradient) followed by 10–45% eluent II in eluent I (45 min gradient).

Low resolution MS (MALDI-TOF): calc. $[C_{382}H_{626}N_{106}O_{116}PS_{4}]^{+}$ $[M + H]^{+}$: 8717.51 Da; found: 8717.22 Da.

HR-MS: calc. $[M + 8H]^{8+}$: 1090.5700 Da; found: 1090.6937 Da

calc. $[M + 9H]^{9+}$: 969.5074 Da; found: 969.6179 Da

calc. $[M + 10H]^{10+}$: 872.6574 Da; found: 872.7562 Da

calc. $[M + 11H]^{11+}$: 793.4164 Da; found: 793.5068 Da

calc. $[M + 12H]^{12+}$: 727.3823 Da; found: 727.3818 Da

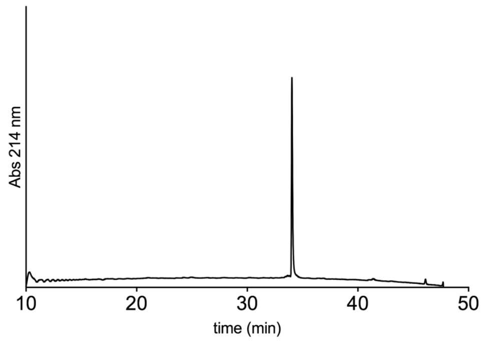

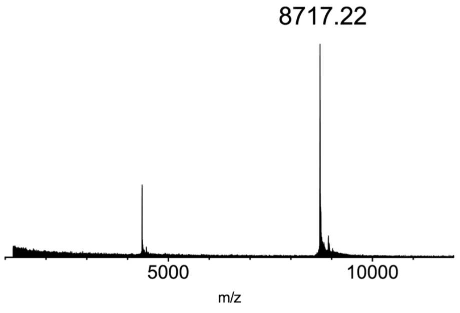

Peptide X$_{Nav1.5\_DIII\text{-}DIV\ Linker\_tAcLys1479}$

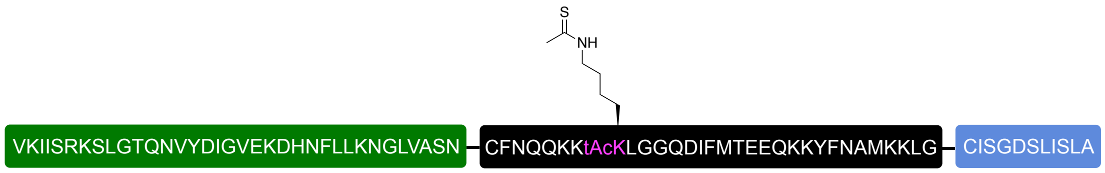

The full peptide was assembled according to the general procedure “N to C directed ligations” described above. For the second ligation, the reaction mixture was incubated at room temperature. Preparative HPLC purification followed by lyophilization yielded the peptide as a fluffy solid (1.2 mg as a TFA salt; yield 20%).

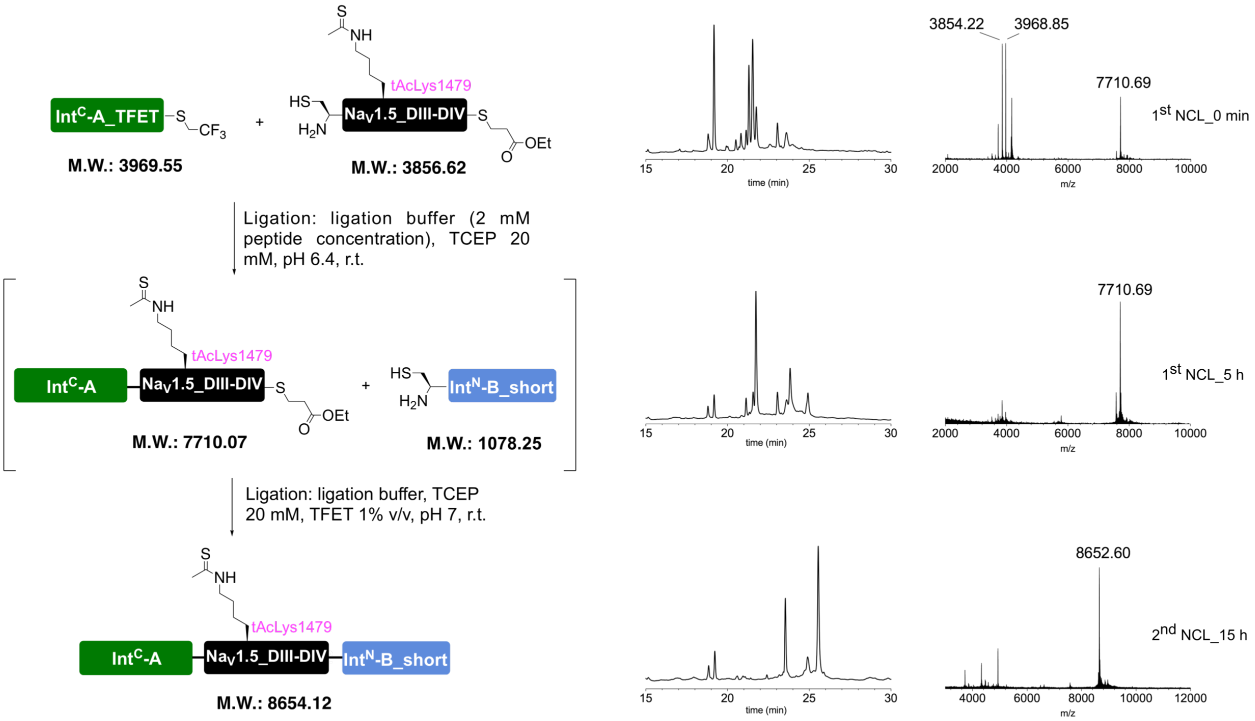

Prep-HPLC purification conditions (C8 column): 0–10% eluent II in eluent I (5 min gradient) followed by 10–45% eluent II in eluent I (45 min gradient).

Low resolution MS (MALDI-TOF): calc. $[C_{383}H_{625}N_{104}O_{113}S_5]^+$ $[M + H]^+$: 8653.51 Da; found: 8653.47 Da.

HR-MS: calc. $[M + 8H]^{8+}$: 1082.5699 Da; found: 1082.6946 Da

calc. $[M + 9H]^{9+}$: 962.3963 Da; found: 962.5064 Da

calc. $[M + 10H]^{10+}$: 866.2573 Da; found: 866.3566 Da

calc. $[M + 11H]^{11+}$: 787.5982 Da; found: 787.6886 Da

calc. $[M + 12H]^{12+}$: 722.0490 Da; found: 722.1322 Da

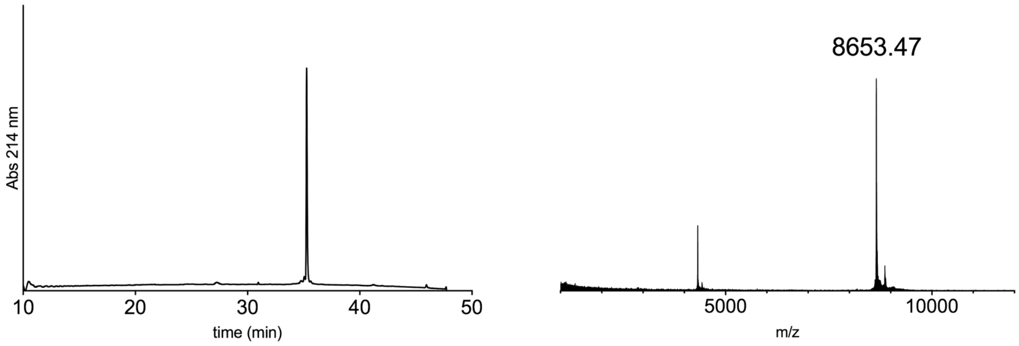

Peptide X$_{Nav1.5_DIII\text{-}DIV\ Linker_tAcLys1479\ +\ phTyr1495}$

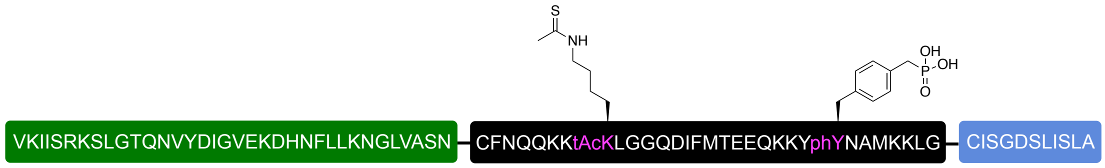

The full peptide was assembled according to the general procedure “N to C directed ligations” described above. Preparative HPLC purification followed by lyophilization yielded the peptide as a fluffy solid (1.2 mg as a TFA salt; yield 20%).

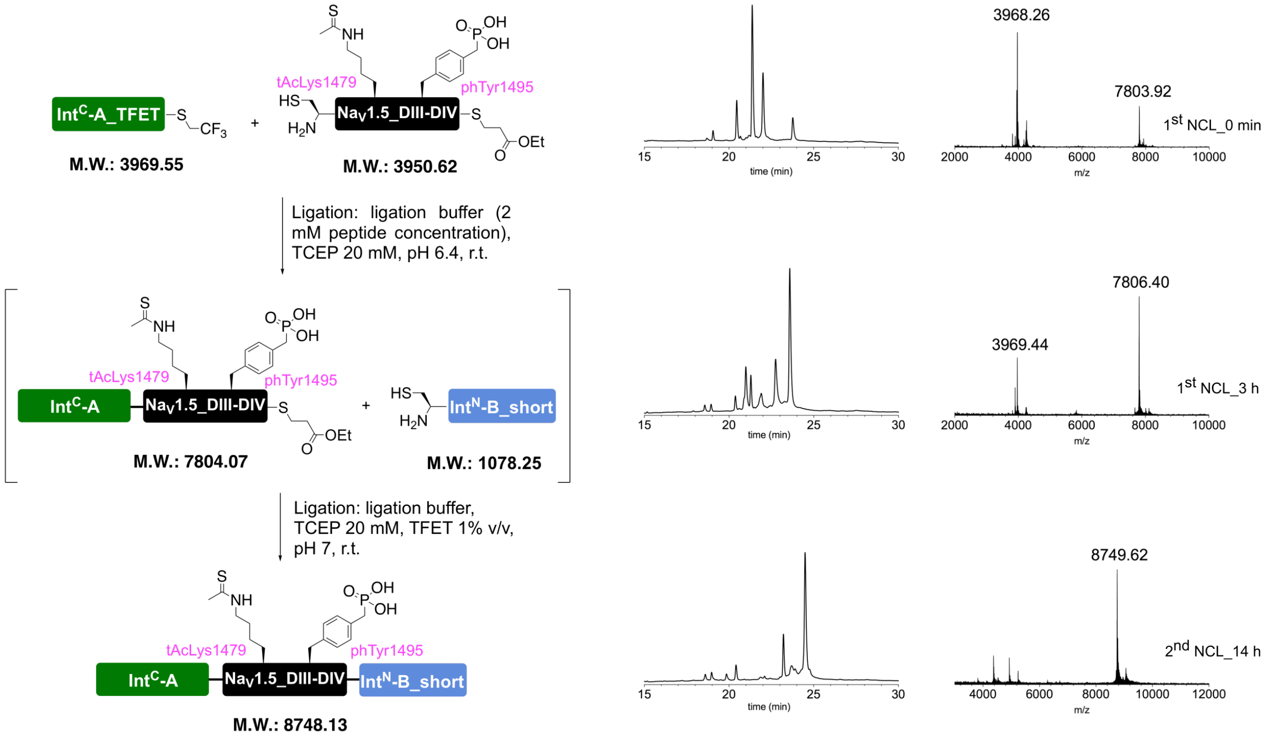

Prep-HPLC purification conditions (C8 column): 0–10% eluent II in eluent I (5 min gradient) followed by 10–45% eluent II in eluent I (45 min gradient).

Low resolution MS (MALDI-TOF): calc. $[C_{384}H_{628}N_{104}O_{116}PS_5]^+$ $[M + H]^+$: 8747.49 Da; found: 8745.23 Da.

HR-MS: calc. $[M + 8H]^{8+}$: 1094.3176 Da; found: 1094.4417 Da

calc. $[M + 9H]^{9+}$: 972.8387 Da; found: 972.9489 Da

calc. $[M + 10H]^{10+}$: 875.6555 Da; found: 875.7544 Da

calc. $[M + 11H]^{11+}$: 796.1420 Da; found: 796.1411 Da

calc. $[M + 12H]^{12+}$: 729.8808 Da; found: 729.9635 Da

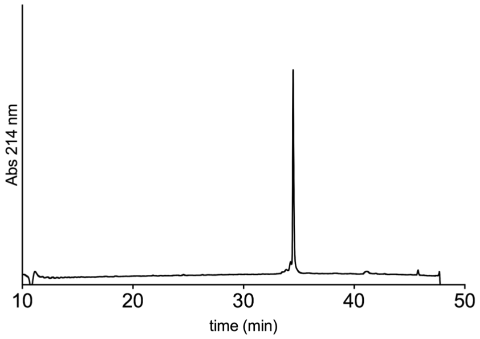

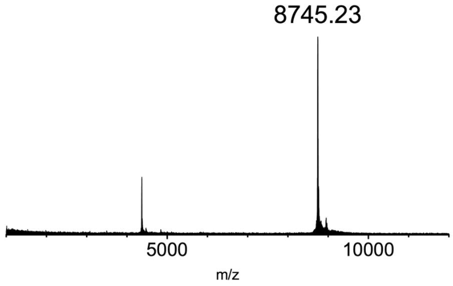

Supplementary References

1) Pless, S.A. *et al.* Asymmetric functional contributions of acidic and aromatic side chains in sodium channel voltage-sensor domains. *J. Gen. Physiol.* **143,** 645-56, doi:10.1085/jgp.201311036 (2014).

2) Gude, M., Ryf, J. & White, P.D. An accurate method for the quantitation of Fmoc-derivatized solid phase supports. *Lett. Pept. Sci.* **9**, 203–206, doi.org/10.1007/BF02538384 (2002).

3) Blanco-Canosa, J.B., Nardone, B., Albericio, F. & Dawson, P.E. Chemical Protein Synthesis Using a Second-Generation *N*-Acylurea Linker for the Preparation of Peptide-Thioester Precursors. *J. Am. Chem. Soc.***137,** 7197-209, doi:10.1021/jacs.5b03504 (2015).

4) Kuzniewski, C.N., Gertsch, J., Wartmann, M. & Altmann, K.H. Total synthesis of hypermodified epothilone analogs with potent in vitro antitumor activity. *Org. Lett.* **10,** 1183-6. doi:10.1021/ol800089x (2008).

5) Gless, B.H. *et al.* Structure-Activity Relationship Study Based on Autoinducing Peptide (AIP) from Dog Pathogen S. schleiferi. *Org. Lett.***19,** 5276-5279. doi: 10.1021/acs.orglett.7b02550 (2017).

6) Watson, E.E. *et al.* Mosquito-Derived Anophelin Sulfoproteins Are Potent Antithrombotics. *ACS Cent. Sci.* **4,** 468-476. doi:10.1021/acscentsci.7b00612 (2018).

7) Beck, W. & Jung, G. Convenient reduction of S-oxides in synthetic peptides, lipopeptides and peptide libraries. *Lett. Pept. Sci.* **1,** 31-37, doi.org/10.1007/BF00132760 (1994).

8) Thompson, R.E. *et al.* Trifluoroethanethiol: an additive for efficient one-pot peptide ligation-desulfurization chemistry. *J. Am. Chem. Soc.* **136**, 8161-4. doi: 10.1021/ja502806r (2014).
