# Supplementary material for: Chemical modification of proteins by insertion of synthetic peptides using tandem protein trans-splicing
Source: Nat Commun. 2020 May 8;11:2284. doi: 10.1038/s41467-020-16208-6 (PMC7210297; doi:10.1038/s41467-020-16208-6)
Supplement: Supplementary file 2 — Reporting Summary [file 41467_2020_16208_MOESM2_ESM.pdf]

Corresponding author(s): Stephan A Pless
Last updated by author(s): Apr 17, 2020

Reporting Summary

Nature Research wishes to improve the reproducibility of the work that we publish. This form provides structure for consistency and transparency in reporting. For further information on Nature Research policies, see Authors & Referees and the Editorial Policy Checklist .

Statistics

For all statistical analyses, confirm that the following items are present in the figure legend, table legend, main text, or Methods section.

n/a	Confirmed	
☐	☒	The exact sample size (*n*) for each experimental group/condition, given as a discrete number and unit of measurement
☐	☒	A statement on whether measurements were taken from distinct samples or whether the same sample was measured repeatedly
☐	☒	The statistical test(s) used AND whether they are one- or two-sided<br>*Only common tests should be described solely by name; describe more complex techniques in the Methods section.*
☒	☐	A description of all covariates tested
☐	☒	A description of any assumptions or corrections, such as tests of normality and adjustment for multiple comparisons
☐	☒	A full description of the statistical parameters including central tendency (e.g. means) or other basic estimates (e.g. regression coefficient) AND variation (e.g. standard deviation) or associated estimates of uncertainty (e.g. confidence intervals)
☐	☒	For null hypothesis testing, the test statistic (e.g. *F*, *t*, *r*) with confidence intervals, effect sizes, degrees of freedom and *P* value noted<br>*Give P values as exact values whenever suitable.*
☒	☐	For Bayesian analysis, information on the choice of priors and Markov chain Monte Carlo settings
☒	☐	For hierarchical and complex designs, identification of the appropriate level for tests and full reporting of outcomes
☒	☐	Estimates of effect sizes (e.g. Cohen's *d*, Pearson's *r*), indicating how they were calculated

Our web collection on statistics for biologists contains articles on many of the points above.

Software and code

Policy information about availability of computer code

Data collection	pClamp10 (Molecular Devices)
Data analysis	ClampFit10 (Molecular Devices); Illustrator 2020 (Adobe); Prism8 (GraphPad) SigmaPlot 13.0 (SPSS); mMass (Open source); Zen 2.3 black (Zeiss); ImageJ/Fiji (open source); ChemDraw 16 (PerkinElmer); FlowJo 10 (Becton Dickinson)

For manuscripts utilizing custom algorithms or software that are central to the research but not yet described in published literature, software must be made available to editors/reviewers. We strongly encourage code deposition in a community repository (e.g. GitHub). See the Nature Research guidelines for submitting code & software for further information.

Data

Policy information about availability of data

All manuscripts must include a data availability statement. This statement should provide the following information, where applicable:
- Accession codes, unique identifiers, or web links for publicly available datasets
- A list of figures that have associated raw data
- A description of any restrictions on data availability

All relevant data collected, analyzed and shown in the figures are available in source data.

Field-specific reporting

Please select the one below that is the best fit for your research. If you are not sure, read the appropriate sections before making your selection.

☒ Life sciences ☐ Behavioural & social sciences ☐ Ecological, evolutionary & environmental sciences

For a reference copy of the document with all sections, see nature.com/documents/nr-reporting-summary-flat.pdf

Life sciences study design

All studies must disclose on these points even when the disclosure is negative.

Sample size	No sample-size calculations were performed, but all electrophysiological experiments were performed on a minimum of 5 cells. The number of recordings was primarily based on the variability of the data, resulting in sample sizes similar to those typically used in the field.
Data exclusions	Electrophysiological recordings with excessive noise or significant amount of endogenous currents at highly de- or hyperpolarised potentials were not considered for analysis. These were pre-established exclusion criteria.
Replication	All experiments were reproduced on at least two batches of cells/cell preparations. All replications were successful.
Randomization	The experiments were not randomized, but wherever possible, experimental groups were recorded in varying order (e.g. WT, Mutant #1, Mutant #2, WT, Mutant #2, Mutant #1 etc)
Blinding	The experimenters were not blinded to allocation during experiments and analysis, but key experiments (electrophysiology and western blots) were repeated by at least two individuals.

Reporting for specific materials, systems and methods

We require information from authors about some types of materials, experimental systems and methods used in many studies. Here, indicate whether each material, system or method listed is relevant to your study. If you are not sure if a list item applies to your research, read the appropriate section before selecting a response.

Materials & experimental systems

n/a	Involved in the study
☐	☒ Antibodies
☐	☒ Eukaryotic cell lines
☒	☐ Palaeontology
☐	☒ Animals and other organisms
☒	☐ Human research participants
☒	☐ Clinical data

Methods

n/a	Involved in the study
☒	☐ ChIP-seq
☐	☒ Flow cytometry
☒	☐ MRI-based neuroimaging

Antibodies

Antibodies used	Anti-P2X2 (#APR-003, Alomone labs); Anti-Nav1.5 [493-511] (#ASC-005, Alomone labs); Anti-Nav1.5 [1978-2016](#ASC-013, Alomone labs); HRP-conjugated goat anti-rabbit secondary antibody (W401B, Promega)
Validation	Anti-P2X2 and Anti-Nav1.5 antibodies were validated for western blot application in the manuscript on recombinant WT proteins (P2X2 or Nav1.5) expressed in Xenopus laevis oocytes. Bands for P2X2 and Nav1.5 constructs were consistent with those stated by the vendor. All antibodies have additionally been validated by the manufacturer and in multiple peer-reviewed publications.

Eukaryotic cell lines

Policy information about cell lines

Cell line source(s)	HEK293 (human embryonic kidney cells), purchased from ATCC.
Authentication	The cell line was not authenticated.
Mycoplasma contamination	The cells were tested negative for mycoplasma contamination.
Commonly misidentified lines (See ICLAC register)	No commonly misidentified cell lines were used in the study

Animals and other organisms

Policy information about studies involving animals; ARRIVE guidelines recommended for reporting animal research

Laboratory animals	Female Xenopus laevis frogs of unknown age (i.e. age not specified by the supplier)
Wild animals	No wild animals were used in this study.

Field-collected samples	This study did not contain samples from the field.
Ethics oversight	Stage V/VI oocytes were obtained from ovaries of female Xenopus laevis frogs (anaesthetized in 0.3% tricaine). This was done under animal license 2014–15-0201–00031, approved by the Animal Experimentation Inspectorate and the Animal Experimentation Council of the Danish Veterinary and Food Administration. Frogs were housed and cared for by a facility approved by the University of Copenhagen.

Note that full information on the approval of the study protocol must also be provided in the manuscript.

Flow Cytometry

Plots

Confirm that:

- [x] The axis labels state the marker and fluorochrome used (e.g. CD4-FITC).
- [x] The axis scales are clearly visible. Include numbers along axes only for bottom left plot of group (a 'group' is an analysis of identical markers).
- [x] All plots are contour plots with outliers or pseudocolor plots.
- [x] A numerical value for number of cells or percentage (with statistics) is provided.

Methodology

Sample preparation	HEK293 cells were grown in Dulbecco's modified Eagle's Medium (DMEM) (Gibco) supplemented with 10 % Fetal Bovine Serum (Biowest), and incubated at 37 ºC with 5 % of CO2. 400.000 cells were seeded in 6-wells plates and incubated for 24 hrs. prior transfection. DNA coding for three GFP-split intein fusion fragments (N, X and C) was co-transfected in a 1:1:1 ratio using a total of 4.5 µg DNA. To keep the same amount of DNA for each combination pcDNA3.1+ empty vector was co-transfected for the N+C and WT eGFP control experiments. Cells were transfected using 6 µg PEI and incubated for circa 44 hrs. The cells were then detached with trypsin-EDTA, spun down and resuspended in PBS containing formaldehyde 37% (1:40) and DAPI 200 µM (1:200). The cell suspension was then passed through a cell-strainer cap and analyzed with BD™ LSR II flow cytometer within 15 min.
Instrument	BD™ LSR II flow cytometer
Software	FlowJo v10 (Becton Dickinson)
Cell population abundance	'GFP-positive' populations<br>Batch#1: eGFP_rec 77.2% (2175/2819 cells); N+X+C 2.66% (151/5683 cells); N+C 0% (0/5471 cells); untransfected 0.08% (2/2635 cells)<br>Batch#2: eGFP_rec 81.4% (16413/20171 cells); N+X+C 5.84% (1359/23257 cells); N+C 0.09% (19/20857 cells); untransfected 0.01% (2/21222 cells)<br>Batch#3: eGFP_rec 49.8% (924/1854 cells); N+X+C 3.86% (143/3706 cells); N+C 0% (0/2157 cells); untransfected 0.34% (12/3545 cells).<br>Purity of the samples was not determined
Gating strategy	The gating strategy was initially based on untransfected samples. The initial population was first gated based on a SSC-A/FSC-A plot, followed by a second gating based on DAPI staining. The sorted cell population was then plotted as number of cells/Alexa 488-fluorescence and visualized as a histogram: the intensity cut off determining GFP positive cells was set at the upper limit of fluorescence distribution from the untransfected cell population. This overall gating strategy was then applied to the other samples of the batch.

- [x] Tick this box to confirm that a figure exemplifying the gating strategy is provided in the Supplementary Information.
